# Prevalence aware feature selection improves biomarker identification in microbiome studies

**DOI:** 10.1093/bioinformatics/btag371

**Published:** 2026-06-24

**Authors:** Ruoxi Yang, Yingjie Li, Kris Sankaran, Thomas A Mace, Phil A Hart, Qin Ma, Xu-Wen Wang, Shanlin Ke

**Affiliations:** Division of Gastroenterology, Hepatology and Nutrition, Department of Internal Medicine, College of Medicine, The Ohio State University Wexner Medical Center, Columbus, OH 43210, United States; Department of Biomedical Informatics, The Ohio State University Wexner Medical Center, Columbus, OH 43210, United States; Department of Statistics, University of Wisconsin—Madison, Madison, WI 53706, United States; Division of Gastroenterology, Hepatology and Nutrition, Department of Internal Medicine, College of Medicine, The Ohio State University Wexner Medical Center, Columbus, OH 43210, United States; The James Comprehensive Cancer Center, The Ohio State University, Columbus, OH 43210, United States; Division of Gastroenterology, Hepatology and Nutrition, Department of Internal Medicine, College of Medicine, The Ohio State University Wexner Medical Center, Columbus, OH 43210, United States; Department of Biomedical Informatics, The Ohio State University Wexner Medical Center, Columbus, OH 43210, United States; Pelotonia Institute for Immuno-Oncology, James Comprehensive Cancer Center, The Ohio State University, Columbus, OH 43210, United States; Channing Division of Network Medicine, Department of Medicine, Brigham and Women’s Hospital, Harvard Medical School, Boston, MA 02115, United States; Division of Gastroenterology, Hepatology and Nutrition, Department of Internal Medicine, College of Medicine, The Ohio State University Wexner Medical Center, Columbus, OH 43210, United States; The James Comprehensive Cancer Center, The Ohio State University, Columbus, OH 43210, United States

## Abstract

**Motivation:**

Identifying robust microbial biomarkers is crucial for disease diagnosis and prediction, elucidation of biological mechanisms, and development of targeted therapies. Machine learning-based approaches, particularly the random forest model, have been widely used for biomarker identification during sample stratification. However, those biomarkers often vary considerably for the same disease, limiting their practical applicability. A robust framework for reliable biomarker identification in microbiome research is needed. To address this gap, we proposed a prevalence-aware feature selection framework (ParSlet) that incorporates a universal scaling relationship between taxon prevalence and selection frequency.

**Results:**

We first identified a universal exponential scaling law linking the probability of a taxon being consistently recognized as a biomarker versus its prevalence. Then, we integrated this scaling law with taxa prevalence into the biomarker identification using random forest. We systematically evaluated this approach in both simulated microbiome datasets and real-world microbiome datasets and compared it with existing methods, finding that our integrated approach generally improved feature stability and reproducibility of biomarker identification. In colorectal cancer (CRC) datasets, our method robustly identified well-established microbial biomarkers such as *Ruminococcus*, *Clostridium_XVIII*, and *Faecalibacterium.* Integrating a prevalence-based scaling adjustment into feature importance enhances the stability of microbiome biomarker identification. This approach holds promise for enabling more reliable disease diagnostics, uncovering generalizable microbial signatures across cohorts, and guiding the development of targeted microbiome-based interventions.

**Availability and implementation:**

ParSlet is available at https://github.com/KelabatOSU/Feature_selection.

## 1 Introduction

Advances in high-throughput sequencing technologies have enabled us to understand human diseases at multiple molecular levels, such as genomics, transcriptomics, proteomics, metabolomics, and microbiome (Karczewski and Snyder 2018). These omics data have fundamentally improved our understanding of the etiology of human diseases (Hasin *et al*. 2017). In particular, microorganisms harbored in or on the human body, including the skin, mouth, lungs, and gut, play essential roles in the development, function, and homeostasis of the human host (Yang *et al*. 2022). They are involved in human health and disease via different mechanisms, such as the immune regulation, nutrient metabolism, and colonization resistance (Ogunrinola *et al*. 2020). Disruption of this balance, or dysbiosis has been linked to many diseases (Dai *et al*. 2022), including *Clostridioides difficile* infection (CDI) (Gonzales-Luna *et al*. 2023, Britton and Young 2014), inflammatory bowel disease (IBD) (Ott *et al.* 2004), asthma (Wang *et al.* 2023), and chronic obstructive pulmonary disease (COPD) (Liang *et al*. 2023).

Given the importance of host-microbiota interactions in human diseases, the microbiome has been increasingly leveraged for the prognostication of clinical outcomes (Barcik *et al*. 2020). For instance, machine-learning analysis of the MicrobiomeHD database has revealed both shared and disease-specific microbial signatures across multiple conditions (Duvallet *et al*. 2017), classified IBD status and subtype from gut microbiome and metabolites (Franzosa *et al*. 2019), and stratified the participants into clinical subgroups of cardiometabolic disease from microbial and plasma metabolome features (Fromentin *et al*. 2022). A broad array of classification models has been used to classify the disease status from the microbiome data, ranging from traditional machine learning algorithms [e.g. random forest, gradient boosting trees, logistic regression, support vector machine (Marcos-Zambrano *et al*. 2021)] to advanced deep-learning approaches (Wang *et al*. 2021, Lee and Rho 2022, Piccolo *et al*. 2022).

A key objective of using classifiers for disease status stratification is to distinguish disease states or subtypes (Karwowska *et al*. 2025). In addition, such classifiers can highlight microbiome taxa or biomarkers that are potentially associated with the disease. For instance, *Ruminococcus gnavus*, which has been associated with IBD, and *Fusobacterium mortiferum*, a species linked to colorectal cancer (CRC), contributed most to disease classification based on gut microbiome data from case–control studies (Sun *et al*. 2024). *Ruminococcus gnavus* and *Clostridium acetireducens* have been identified as key microbial biomarkers for colorectal cancer (CRC) and hepatocellular carcinoma (HCC) subtypes (Kolisnik *et al*. 2023). Those biomarkers can suggest potential underlying biological mechanisms, support hypothesis generation, and serve as candidates for therapeutic targets. However, they can vary substantially across different studies for the same disease. This inconsistency limits their practical applicability and interpretability. Moreover, current classifiers often lack reproducibility across datasets and may identify taxa that are dataset-specific rather than truly disease-related. Given the increasing adoption of classification-based analyses in microbiome research, the development of a robust approach for microbiome biomarker identification has the potential to significantly advance the field by providing reproducible and generalizable tools for discovery.

Building on insights from our bibliometric analysis, which highlighted reproducibility as a persistent gap in microbiome biomarker studies, we present a user-friendly computational framework (Fig. S1, available as supplementary data at *Bioinformatics* online), by leveraging a universal scaling law between the probability of a species being consistently recognized as a biomarker versus its prevalence (i.e. the proportion of samples in which the taxon was present). Then, we integrated this scaling law into the biomarker identification using the random forests. We systematically evaluated our approach in both simulated datasets and various real-world datasets, finding that the integrated approach consistently outperformed traditional methods in the stability of biomarker identification.

## 2 Materials and methods

### 2.1 Literature analysis

We conducted a literature search in the Web of Science database to assess publication trends in microbiome-based disease classification using random forest models. The timeframe was set from 2010 to 2025. All retrieved publications were exported with metadata, including publication year and keywords. For each record, we manually extracted keywords related to feature importance or biomarker reporting from the title, abstract, and author keywords. Synonymous terms were standardized to canonical categories, including “biomarker” (e.g. “biomarkers,” “marker,” “signature”), “feature importance” (e.g. “variable importance,” “importance score”), and “feature selection” (e.g. “important features,” “feature ranking”). Publications without relevant keywords were excluded from the keyword frequency analysis. We then calculated (i) the annual number of publications meeting the search criteria and (ii) the frequency distribution of standardized keywords. Annual publication counts were visualized as bar plots, and keyword frequencies were visualized using a treemap. All analyses were performed in R using the packages dplyr, tidyr, ggplot2, and treemap.

### 2.2 Prevalence-aware feature selection framework

We propose ParSlet, a prevalence-aware feature selection framework that incorporates taxon prevalence into random forest feature importance to improve the stability and reproducibility of biomarker identification. Taxon prevalence is defined as the proportion of samples in which a given taxon is present and is computed using the training data only. Feature importance scores obtained from Random Forest models (Gini impurity and mean decrease accuracy) are adjusted by scaling them with prevalence raised to a power exponent α. In this study, α=2.5 was selected based on model fitting performance in simulated data.

Algorithm 1 summarizes the proposed prevalence-aware feature selection framework.

**Algorithm 1: Prevalence-aware feature selection (ParSlet)** 

 **Input**: Feature matrix $X\in R^{n\times p}$ with n samples and p features, outcome vector $y\in R^{n}$, prevalence exponent α, number of selected features k

 **Output**: Selected feature set S

1 **Compute prevalence for each feature:** 

2  for j = 1 to p do

3   prev[j] ← (1/n) * sum over i = 1 to n of I(X[i,j] > 0)

4  end for

5 **Train a Random Forest model using X and y**:

6  model ←Fit(RandomForest, X, y)

7 **Obtain raw importance**  imp [j]  **for each feature j**:

8  imp [j]←Importance (model, j)

9 **Adjust importance by prevalence**:

10  $\mathrm{score}\;[j]\leftarrow \mathrm{imp}\;[j]*\mathrm{prev}\;{\left[j\right]}^{\alpha }$

11 **Rank all features in descending order according to**  score [j]

12 **Select the top k ranked features** 

13 **return S** 

### 2.3 Synthetic microbiome data

Synthetic microbiome data were generated by SparseDOSSA (Ma *et al*. 2021) using the function SparseDOSSA2 with the stool template. The number of features in simulated data is the same as the original data, and the spike-in configuration is set to “both” (taxa’s abundance and prevalence). We generated simulated data by varying the percentage of features associated with metadata and the effect size of metadata, representing the log fold change for abundance spike-in and log odds ratio for prevalence spike-in.

Synthetic microbiome data were generated using the SparseDOSSA2 function from the SparseDOSSA2 R package (template = “Stool”). The number of features in the simulated data was kept identical to the template. Associations between binary metadata (“Health” versus “Nonhealthy”) and taxa were introduced by setting spike_metadata to "both", so that spiked features differed in both abundance and prevalence. We varied the proportion of taxa associated with metadata (5%, 10%, 20%) and the metadata effect size, corresponding to log fold changes for abundance and log odds ratios for prevalence. Sample sizes were set to 10%–50% of the 332 template samples, in increments of 5%. For each parameter combination, we saved both the simulated relative abundance table and the corresponding ground-truth spike-in feature list.

We evaluated the stability of feature selection in simulated microbiome datasets across varying sample sizes and fractions of spiked-in differentially abundant taxa ($f_{D}$ = 0.05, 0.1, 0.2). For each simulated dataset, a random forest classifier was trained to predict a binary case/control phenotype, and feature importance was computed using Gini impurity and mean decrease accuracy, respectively. The top 10% of taxa ranked by each importance metric were retained as candidate biomarkers. To quantify stability, we calculated the average pairwise overlap between top feature sets obtained from models trained on different normalized sample sizes ($f_{S}$), defined as:


(1) $$\mathrm{Overlap}=\frac{{|F}_{i}\cap F_{j}|}{k}$$

where $F_{i}$ and $F_{j}$ are the selected features set from two sample sizes, and k is the number of selected features. The overlap was averaged over 10 independent simulation replicates for each condition. We compared the original importance rankings (unadjusted) to prevalence-integrated rankings, where importance scores were scaled by taxon prevalence (τ), calculated from the training data only, using either power-scaled integration ($\mathrm{importance}\times \tau^{2.5})$ and linear-scaled integration (importance×τ). Here, prevalence (τ) was defined as the proportion of samples in which the taxon was present. Overlap values were computed separately for unadjusted Gini, unadjusted mean decrease accuracy, prevalence-integrated Gini, and prevalence-integrated mean decrease accuracy rankings. Stability results are presented as the mean ± standard deviation of pairwise overlap across $f_{S}$ values for each $f_{D}$ setting.

### 2.4 Real microbiome data

We download the processed microbiome data from the MDAS (Nearing *et al*. 2022) (rarefied). For the datasets in MDAS, we excluded the studies in which the total sample size was <50, or the samples in each group were <20, and the genera present in <5% of the samples were filtered. Note that we used different sample size thresholds to filter out studies so that fewer studies were excluded, and the samples in each group are sufficient to perform 5-fold cross-validation. The species-level taxonomic profile and labels of curated data were downloaded from the R package curatedMetagenomicData (Pasolli *et al*. 2017). We only retained the phenotypes with multiple studies and a large sample size, which resulting in five disease categories: colorectal cancer (CRC, 5 studies), inflammatory bowel disease (IBD, 4 studies), impaired glucose tolerance (IGT, 2 studies), type 2 diabetes (T2D, 4 studies), and colorectal adenoma (adenoma, 4 studies). In total, these datasets comprised 19 independent cohorts generated using Illumina HiSeq, Illumina NextSeq, and Ion Proton platforms across North America, Europe, and Asia. Baseline characteristics, including median age and BMI, are summarized in Table S2, available as supplementary data at *Bioinformatics* online.

We assessed the stability of feature selection across different sample sizes in ten real microbiome datasets. For each dataset, we used the feature importance values obtained from random forest models (Gini impurity and mean decrease accuracy), as described above. Two ranking approaches were compared: (i) the original importance scores (unadjusted), and (ii) prevalence-integrated scores, where each importance score was multiplied by the taxon prevalence (fraction of samples in which the taxon was present) raised to the power of 2.5.

For each dataset, we considered ten training sample fractions ranging from 0.1 to 1.0 in equal increments. Within each sample fraction, we identified the top 10% most important features according to each ranking method. We then computed the pairwise overlap between the top features from each sample fraction and those from all other sample fractions. The average pairwise overlap, and its standard error were calculated across 10 replicate runs for each fraction.

### 2.5 Classification model

The classification model, random forest, was implemented using the R package randomForest (Liaw and Wiener 2002). The microbiome data were taken as a centered log-ratio transformation by adding a small constant $10^{-10}$. All hyperparameters of the random forest were used with the default setting.

We trained random forest classifiers to distinguish between sample groups in both real and simulated microbiome datasets. For each dataset, we evaluated classification performance across ten training sample fractions ranging from 10% to 100% of available samples. For real datasets, the maximum fraction was restricted to ensure a non-empty test set. For each fraction, we performed 10 independent replicates using different random seeds. In each replicate, samples were randomly partitioned into a training set and a held-out test set. Feature importance was extracted as Gini impurity and mean decrease accuracy. For prevalence-adjusted rankings, each importance score was multiplied by the corresponding taxon’s prevalence (fraction of samples in which the taxon was detected) raised to the power of 2.5.

### 2.6 Statistical test

We performed Wilcoxon rank-sum tests for each taxon to compare two sample groups. The differentially abundant taxa were considered as those taxa and genes with *P*-values <0.001.

## 3 Results

### 3.1 The rising interest in microbiome-based disease stratification

**Figure 1:**
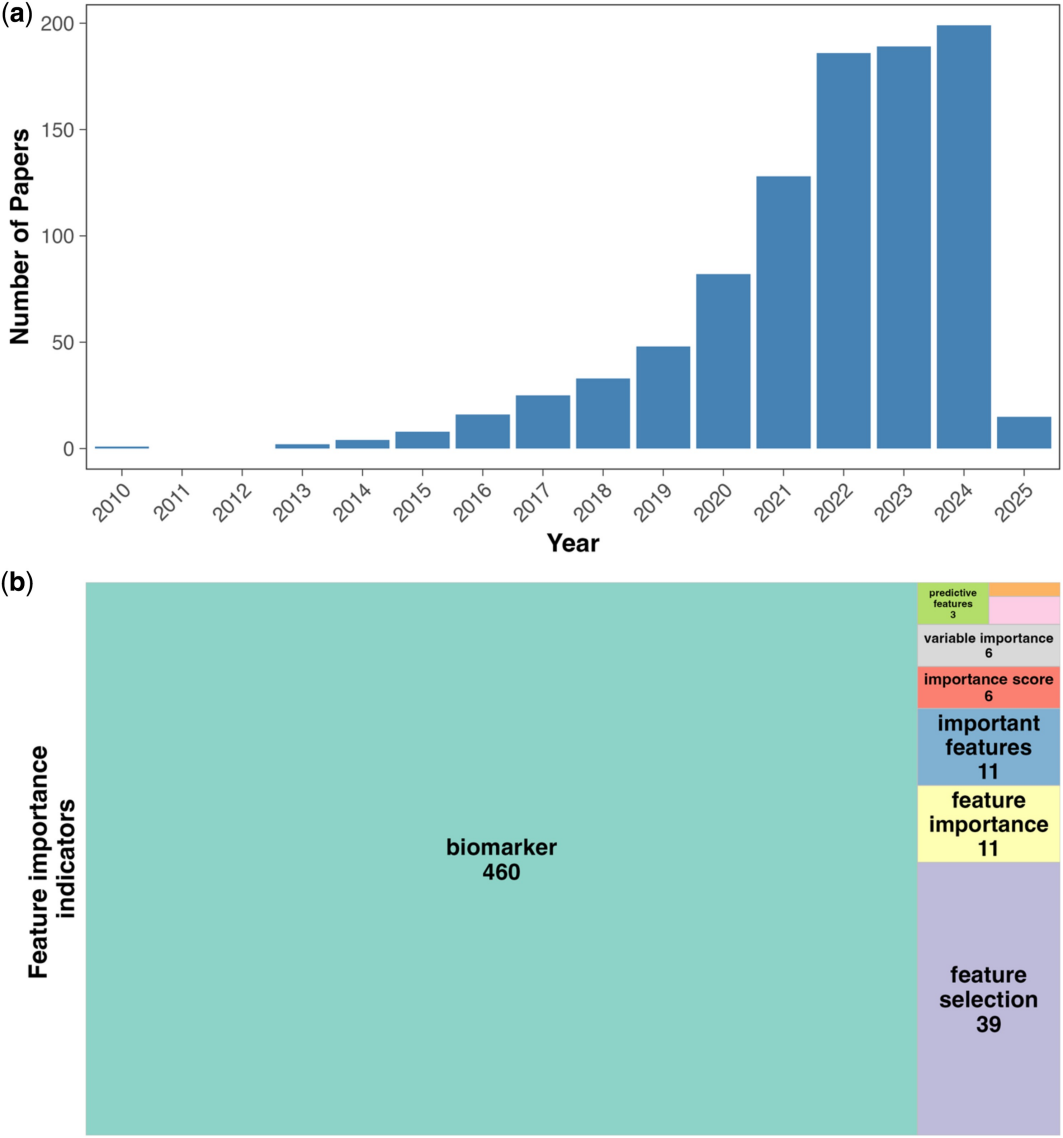
Random forests for microbiome classification and feature selection. (a) A bibliometric search of the Web of Science database was conducted using the keywords “microbiome” AND “random forest.” (b) Feature importance descriptions were subsequently extracted using ChatGPT (extracted as of Mar 2025).

We identified a growing trend in the application of machine learning methods to microbiome-based disease classification and biomarker identification. To better understand this landscape, we conducted a literature analysis using the Web of Science. Our results showed a steep increase in the number of microbiome studies using random forests over the past decade (Fig. 1a), highlighting the popularity of this approach. For instance, the number of publications rose from a handful in 2010 to nearly 200 by 2024. When we examined how these studies reported feature importance, we found that most of them broadly referred to “biomarkers”, with only a small fraction explicitly mentioning terms like “feature selection”, “feature importance”, or “important features” (Fig. 1b). This suggests that while identifying biomarkers is a common goal, many studies may overlook the specific strategies for selecting robust and reproducible features, contributing to the variability seen in predictive performance.

**Figure 2:**
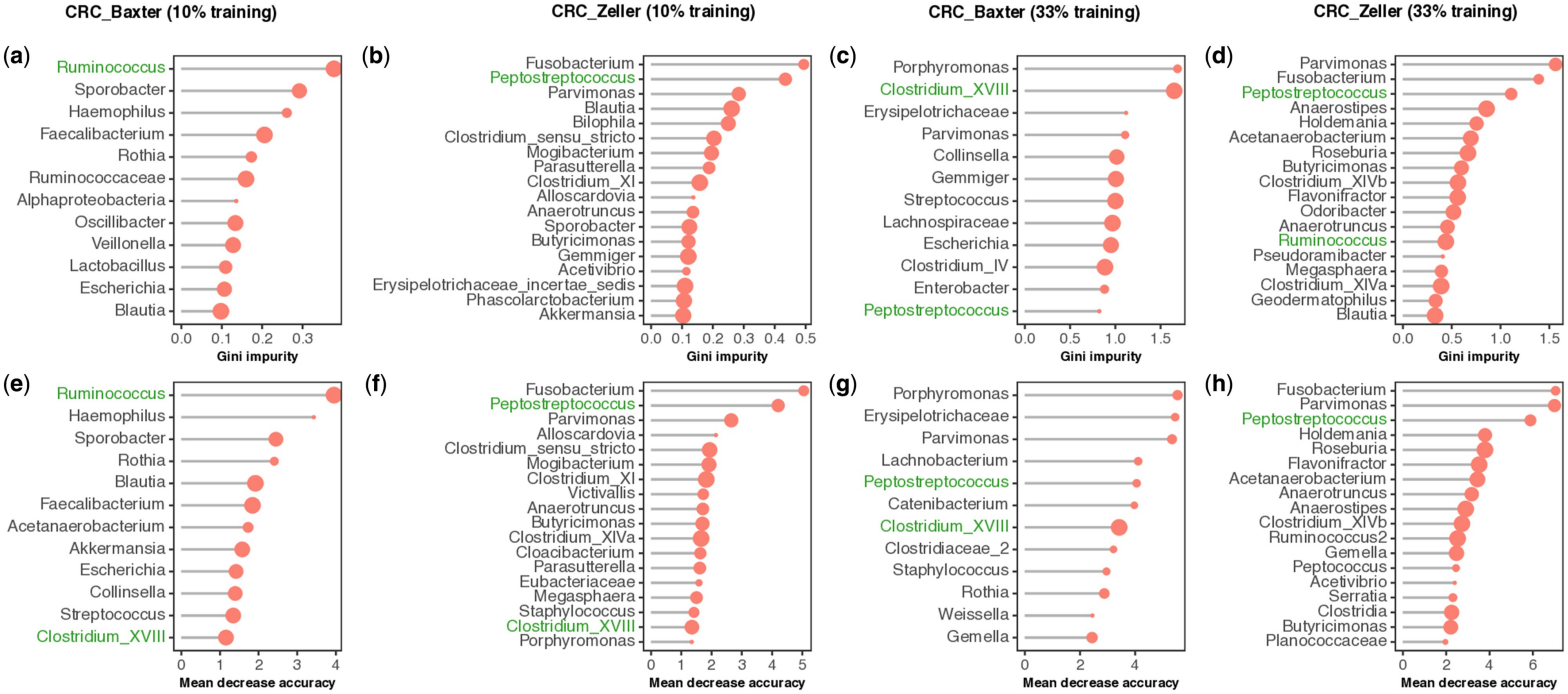
Top 10% of important features identified by random forest from two CRC datasets. Panels a–d and e–h show features ranked by traditional importance metrics (Gini impurity and mean decrease in accuracy, respectively). Marker sizes are proportional to taxa prevalence in the training data. Genera consistently identified as highly important by both ranking strategies across the two datasets, such as Ruminococcus, Clostridium_XVIII, Fusobacterium, Parvimonas, and Peptostreptococcus, are known to be associated with CRC and were robustly selected across different sample sizes and importance criteria, suggesting their potential as reproducible microbial biomarkers for colorectal cancer.

**Inconsistent biomarkers across studies of the same disease.** To examine the consistency of biomarker identification across sample sizes and studies, we trained a random forest model to predict if a microbiome sample is from CRC or healthy control using two CRC datasets: Baxter colorectal cancer (CRC) dataset (120 CRC cases and 172 controls) (Baxter *et al*. 2016) and ZellerG_2014 (53 CRC cases and 61 controls) (Zeller *et al*. 2014), respectively (Fig. 2). For each dataset, we selected the top 10% most important microbial features (based on Gini impurity and mean decrease in accuracy, respectively) using random forest models trained on subsets of the data corresponding to training fractions of 0.1 and 0.33 (i.e. 10% and 33% of the samples, respectively) (Fig. 2a–h). We then compared microbial features from two studies and two training fractions for model training, and we found that the most important features share very few overlaps. For instance, only *Clostridium_XVIII* and *Ruminococcus* were consistently identified from the Baxter CRC study at a training ratio of 0.1 and 0.3, respectively (green, Fig. 2a and b). When we compared those taxa among the two studies, we found that *Clostridium_XVIII* was the only feature consistently identified by the two studies. These findings underscore the critical importance of developing a more robust approach for biomarker identification.

### 3.2 A scaling law linking taxon prevalence to biomarker robustness

The results from previous CRC studies imply that a taxon’s prevalence serves as a critical factor in determining its importance (Wu *et al*. 2009). To examine whether taxa’s prevalence is associated with the robustness as a key biomarker, we generated the synthetic dataset using the SparseDOSSA (Ma *et al*. 2021) (Sparse Data Observations for the Simulation of Synthetic Abundance). SparseDOSSA is a statistical model of microbial ecological population structure that can parameterize real microbial community profiles and simulate new and realistic profiles. In addition, SparseDOSSA can simulate a spiked-in association between microbial features and a synthetic case/control variable. We generated stool-like datasets with varied normalized sample sizes (by the number of taxa) $f_{S}$ and the fraction of differently abundant taxa $f_{D}$ (see Section 2). Then, a random forest classifier was applied to classify the case-control status using the centered log-ratio transformed taxonomic profile. The classification was evaluated using 5-fold cross-validation. We used Gini impurity and mean decrease in accuracy to rank microbial taxa and selected the top 10% taxa as candidate biomarkers. To avoid data leakage, species prevalence was computed from training data only.

**Figure 3:**
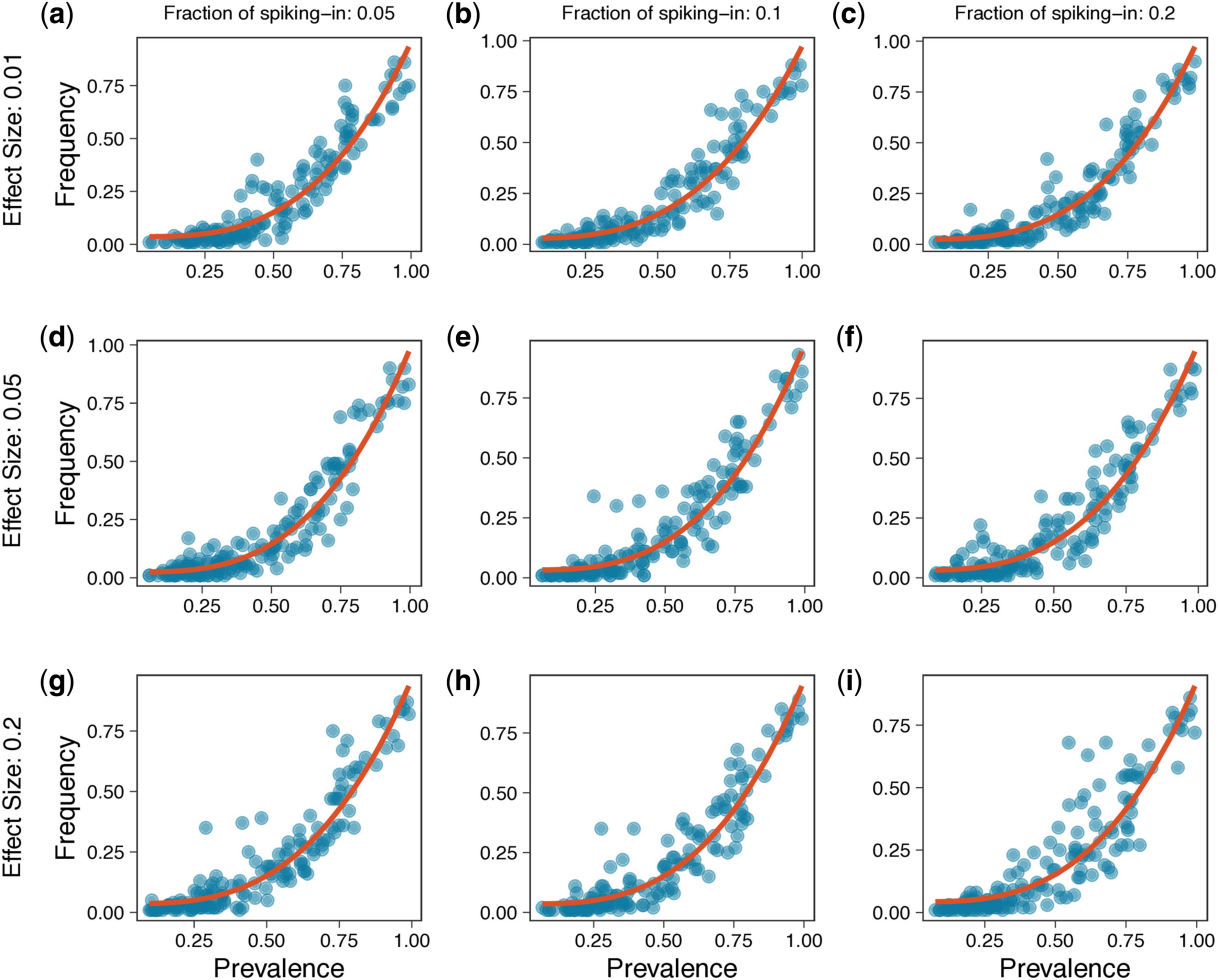
Frequency of a feature’s inclusion among the top 10% most important features versus its prevalence. Each point represents a taxon, showing its average prevalence across simulated samples (*x*-axis) and the frequency with which it was selected among the top 10% most important features across all simulation runs (*y*-axis). Data are shown for simulated microbiome datasets with different fractions of spiked-in taxa ($f_{D}=0.05,\;0.1,\;0.2$; panels a-i). A nonlinear positive association is observed in all settings, indicating that more prevalent features tend to be selected more frequently, although the strength of this association does not markedly increase with higher $f_{D}$.

To investigate how taxon prevalence influences its likelihood of being identified as a biomarker, we analyzed simulated microbiome data generated with varying fractions of spiked-in differentially abundant taxa ($f_{D}=0.05,\;0.1,\;0.2$). For each simulation, we computed the frequency (p) with which each taxon appeared in the top-ranked subset across all runs and its prevalence (τ), defined as the proportion of samples in which the taxon was present. As shown in Fig. 3a–i, we observed a consistent exponential relationship between species prevalence versus the frequency across all levels of spiking-in, e.g. $p\propto \tau^{\alpha }$, which reflects the scaling law described in this section. Based on a systematic comparison of candidate exponent values (*α* = 1–3), we selected *α* = 2.5 as it consistently achieved the lowest Root-Mean-Square Error (RMSE) and the highest *R*^2^ across different effect sizes and spike-in fractions (Fig. S2, available as supplementary data at *Bioinformatics* online). More importantly, this relationship remained relatively stable across increasing $f_{D}$ values, suggesting that prevalence has a persistent influence on feature selection that is not strongly modulated by signal strength.

### 3.3 The prevalence scaling law provided robust biomarker identification in simulated microbiome data

**Figure 4:**
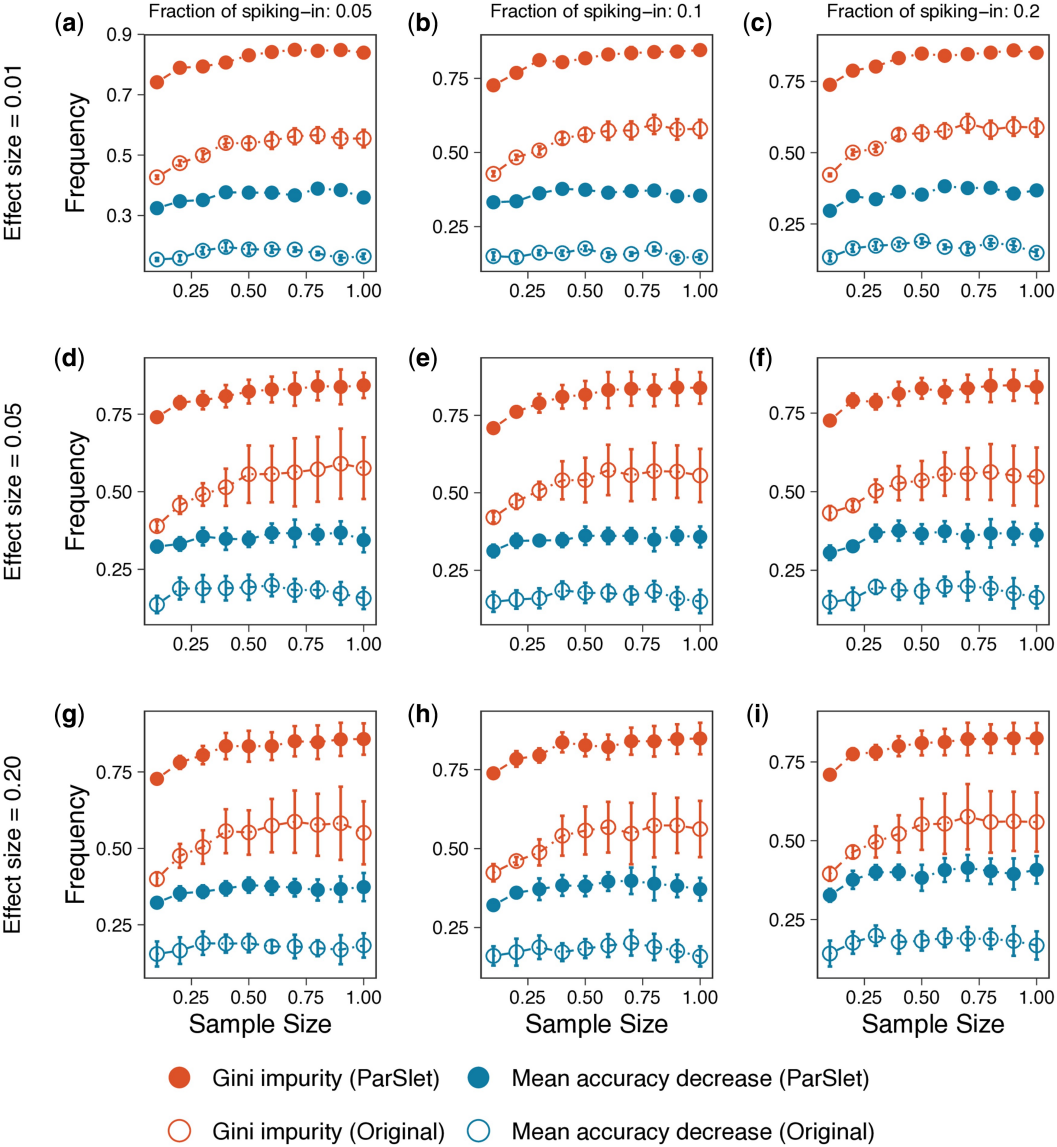
Mean overlap of top 10% selected features across normalized sample sizes and spiking-in fractions using ParSlet. The average pairwise overlap of the top 10% most important features is shown across normalized sample sizes ($f_{S}$) under varying fractions of spiked-in taxa ($f_{D}=0.05,\;0.1,\;0.2$; panels a–i). Feature importance was ranked using either Gini impurity or Mean Decrease Accuracy, with or without prevalence integration (importance × richness^2.5^). ParSlet rankings consistently show higher overlap than unadjusted rankings, suggesting improved feature selection stability across runs.

Then, we examined whether this scaling law can improve the robustness of biomarker identification. To evaluate the stability of selected features across different sample sizes, we computed the average pairwise overlap for each sample size with all other remaining sample sizes of the top 10% most important features ranked by two approaches: (i) Gini impurity or Mean Decrease Accuracy from random forest models without considering the taxa’s prevalence (unadjusted). (ii) An integrated metric, where Gini impurity or Mean Decrease Accuracy was scaled by prevalence raised to the 2.5 power (prevalence-integrated). We performed this analysis on simulated microbiome data with varying fractions of spiked-in differentially abundant taxa ($f_{D}=0.05,\;0.1,\;0.2$). As shown in Fig. 4a –i, we found that across all settings the frequency of overlapping features was consistently higher for prevalence-integrated rankings than for unadjusted ones, suggesting improved stability and reproducibility of feature selection across simulation runs. Furthermore, the increased feature selection stability is robust against sample size and varying fractions of spiked-in taxa. Notably, the integrated Gini-based ranking exhibited the highest overlap across conditions. These findings suggest that incorporating exponentially scaled prevalence into feature ranking can enhance selection robustness, especially under high signal or large sample size scenarios. More importantly, we compared the performance using linear-scaled prevalence in feature importance ranking, finding that the improved frequency is smaller than using exponential-scaled prevalence (see Fig. S3, available as supplementary data at *Bioinformatics* online). We evaluated multiple alternative functional forms for modeling the relationship between feature prevalence and selection frequency, including higher-order polynomials (John Lu 2010) (Fig. S4, available as supplementary data at *Bioinformatics* online), generalized additive models (Hastie and Tibshirani 1986) (Fig. S5, available as supplementary data at *Bioinformatics* online), logistic curves (Cox 1958) (Fig. S6, available as supplementary data at *Bioinformatics* online), and spline-based regressions (Reinsch 1967) (Fig. S7, available as supplementary data at *Bioinformatics* online), and compared them with the proposed power-law adjustment using R^2^. All models demonstrated comparable explanatory performance across different effect sizes and spiking-in fractions, with no single functional form consistently outperforming the others. Given this equivalence, we adopted the power-law formulation because it offers a parsimonious and analytically interpretable representation with fewer parameters across datasets.

To assess the sensitivity of our framework to model choice and hyperparameter settings, we further evaluated a wide range of random forest configurations (Figs S8–S11, available as supplementary data at *Bioinformatics* online). Across different parameter settings, the prevalence-aware feature ranking consistently improved feature selection stability.

We further compared our ParSlet approach with several alternative methods, including LASSO (Tibshirani 1996) (Fig. S12, available as supplementary data at *Bioinformatics* online), Elastic Net (Zou and Hastie 2005) (Fig. S13, available as supplementary data at *Bioinformatics* online), Relief (Kira and Rendell 1992) (Fig. S14, available as supplementary data at *Bioinformatics* online), mRMR (Peng *et al*. 2005) (Fig. S15, available as supplementary data at *Bioinformatics* online), and the prevalence-integrated method PreLect (Chen *et al*. 2025) (Fig. S16, available as supplementary data at *Bioinformatics* online), under the same simulation and subsampling framework. All methods were evaluated using identical training and test splits and the same criteria for stability. Across all simulation settings, our prevalence-aware feature ranking consistently improved feature stability. This trend remained consistent when comparing against PreLect under multiple λ configurations with step sizes of 10, 30, and 50 (Figs S16–S18, available as supplementary data at *Bioinformatics* online).

### 3.4 The scaling law improved the robustness of biomarker identification in real microbiome data

**Figure 5:**
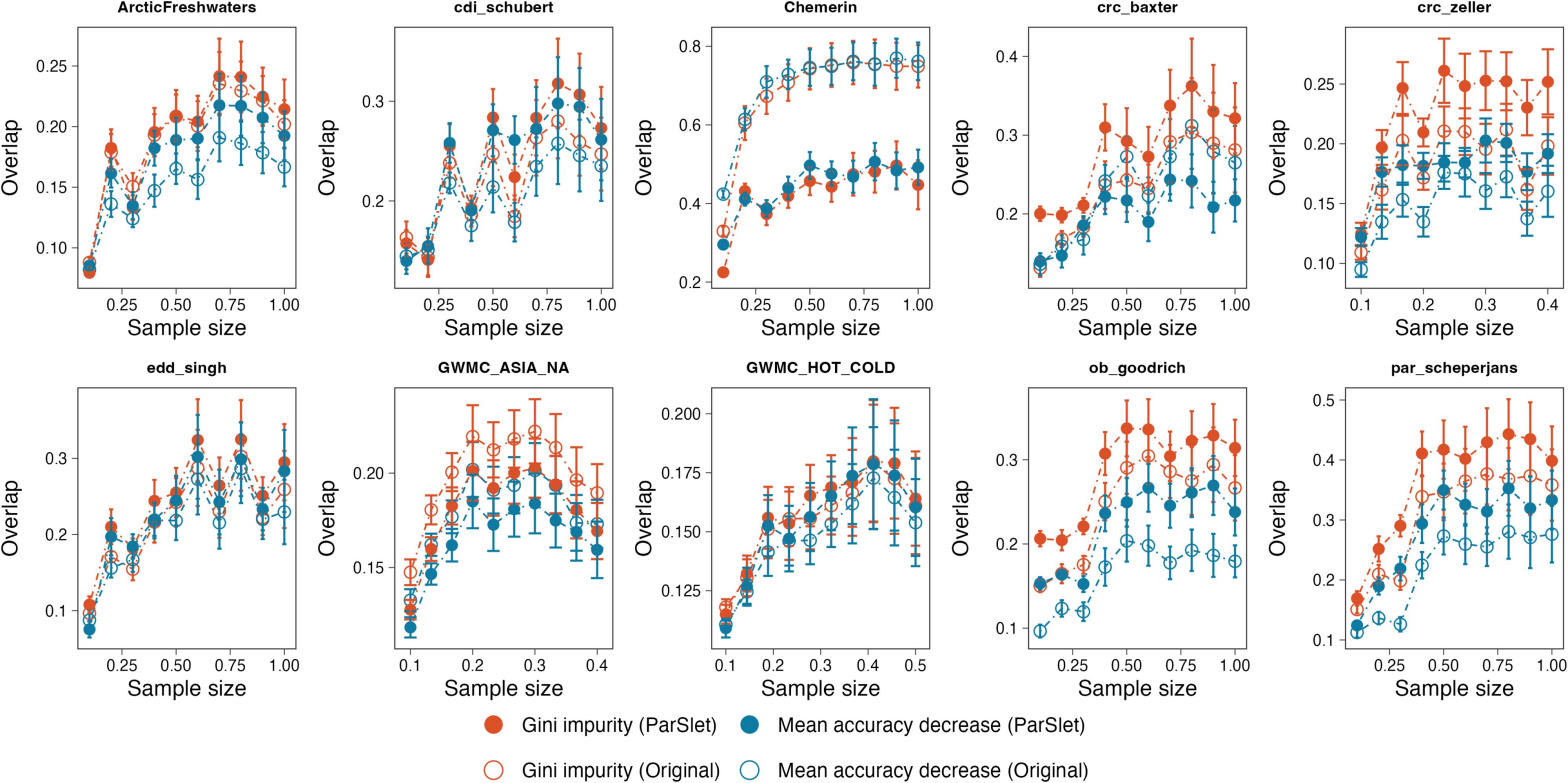
Mean feature overlap across sample fractions in MDAD (Microbiome different abundance datasets) using ParSlet. ParSlet importance measures (Gini impurity and Mean accuracy decrease), which incorporate both feature importance and prevalence, exhibit higher overlap across different sample fractions compared to their original counterparts. This effect becomes more pronounced at larger sample sizes, suggesting that integrating scaled feature prevalence improves the stability and reproducibility of selected biomarkers.

Next, we applied the previously identified scaling law to real microbiome datasets. We used MDAD (Microbiome different abundance datasets) that collected 38 16S rRNA gene datasets with two sample groups (Nearing *et al*. 2022), and we used 10 in total after excluding datasets with sample sizes <50 and those with very imbalanced disease status labels (see Section 2). To assess the stability of feature selection across varying sample sizes, we also computed the pairwise overlap of the top 10% most important features based on both Gini impurity and mean accuracy decrease from random forest models, respectively (shown in Fig. 5). We compared the original importance scores to prevalence-weighted (integrated) versions, using an exponential scaling of prevalence (τ^2.5^). As shown across ten real microbiome datasets, the integrated importance metrics consistently yielded higher overlap scores than the original metrics in eight out of ten, indicating more stable and reproducible feature selection by integrating the exponential scaling law. In particular, the two CRC datasets also provide a clear example of this improvement. Several taxa, such as *Ruminococcus*, *Clostridium_XVIII*, and *Faecalibacterium*, were more frequently identified as important features by ParSlet compared to other methods (Table S1, available as supplementary data at *Bioinformatics* online). Those taxa have consistently been considered CRC biomarkers. For instance, *Faecalibacterium*, which is associated with inflammatory conditions, is diminished in several diseases, including CRC (Martín *et al*. 2023). These shifts highlight how the scaling law refines biomarker identification by reducing spurious taxa and emphasizing those with stronger prevalence support. This effect was particularly pronounced at larger sample sizes, where the integrated Gini and accuracy metrics provided a clear advantage. These results support the hypothesis that exponential-scaled prevalence improves feature selection stability and reproducibility in real microbiome datasets as well. To extend the analysis from feature stability to predictive correctness, we performed cross-cohort validation in independent CRC microbiome studies by training models in one cohort and evaluating them in the remaining cohorts using cross-study AUROC (Fig. S19a, available as supplementary data at *Bioinformatics* online). We found that incorporating prevalence weighting preserves predictive performance while achieving higher AUROC scores, confirming the robustness and generalizability of our approach. Although the difference between the high- and low-prevalence feature groups was not statistically significant based on the corrected resampled *t*-test^37^ (*P* = 0.76), the high-prevalence group exhibited a higher median AUROC than the low-prevalence group (0.64 versus 0.57). In addition, high- and low-prevalence feature groups were confirmed to represent significantly distinct prevalence regimes based on prevalence distribution comparisons across cohorts (Fig. S19b, available as supplementary data at *Bioinformatics* online). To further evaluate stability across datasets without within-dataset resampling using simulated data, we trained the model on all samples from each dataset and applied ParSlet and PreLect for feature selection. Across simulated settings, ParSlet achieves higher feature-selection stability than PreLect in most pairwise comparisons of effect sizes, as measured by the Jaccard similarity of top-selected features (Fig. S20, available as supplementary data at *Bioinformatics* online). In addition, ParSlet shows higher cross-study overlap than PreLect across three independent CRC cohorts, with greater pairwise feature overlap in 4 of 6 comparisons based on the top 5% and 10% ranked features (Fig. S21, available as supplementary data at *Bioinformatics* online).

In the Chemerin dataset, the exponentially scaled feature importance ranking produced an unstable result. To investigate why this occurred, we analyzed the prevalence distribution of taxa across datasets. We found that the microbiome composition in this study was characterized by a lack of sparsity, e.g. most taxa were present in nearly all samples. This pattern contradicts a fundamental property of microbiome data: its inherent sparsity, where the majority of taxa exhibit low prevalence (Fig. S22, available as supplementary data at *Bioinformatics* online).

### 3.5 The prevalence scaling law efficiently increased the true positive rate and decreased the false positive rate

**Figure 6:**
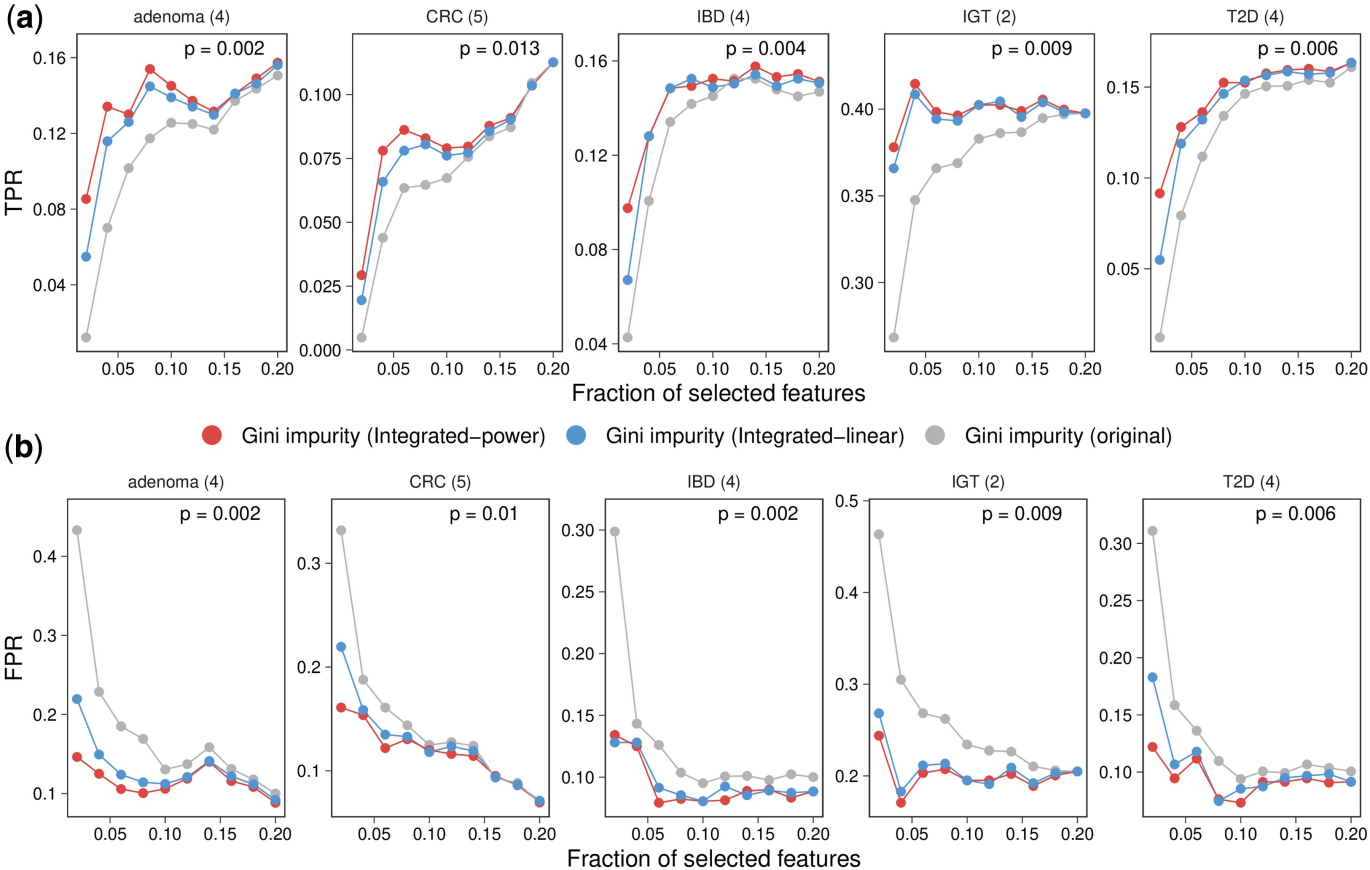
Comparison of integrated and original Gini impurity feature importance in biomarker identification of the diseases across studies. Each panel represents a disease phenotype (colonic adenoma, CRC, IBD, IGT, T2D) using multiple metagenomic studies. (a) shows the true positive rate (TPR) and (b) shows the false positive rate (FPR) as a function of the fraction of selected features. Three versions of Gini importance are compared: original, integrated with linear prevalence adjustment, and integrated with power prevalence adjustment. *P*-values reflect Wilcoxon rank-sum tests comparing integrated (both power-scaled and linear-scaled) versus original across studies and were not adjusted for multiple comparisons.

Finally, we evaluated the impact of integrating taxon prevalence into biomarker identification across studies for the same disease. We collected curated metagenomic data (Nearing *et al*. 2022) across five disease phenotypes (due to multiple studies available for each of those diseases): asthma, CRC, IBD, impaired glucose tolerance (IGT), and type 2 diabetes (T2D). To assess the robustness of biomarkers for a disease, we consider a taxon as a true positive if it is consistently ranked as the top-f most important feature in all studies. Similarly, a taxon was considered a false positive if it is only showing in one study. We found that, compared to the original Gini impurity importance and integration using linear scaled prevalence, the exponential scaled method consistently achieved higher true positive rates (Fig. 6a) and lower false positive rates (Fig. 6b) across a range of selected feature fractions f (Wilcoxon test, *P* < 0.05). This effect was especially evident in phenotypes such as colonic, adenoma, IBD, and T2D, where integrating scaled prevalence led to more stable and accurate feature selection across studies. This highlights the benefit of incorporating prevalence-informed scaling into the feature selection process.

## 4 Discussion

The growing application of AI in microbiome research has enabled integrative analyses of molecular biomarkers and their associations with clinical outcomes. This has enabled us to examine the association of molecular biomarkers associated with clinical outcomes in multiple layers, e.g. genomics, epigenomics, transcriptomics, proteomics, metabolomics, and microbiome (Babu and Snyder 2023). Machine learning models are being used for classification, subtyping, and biomarker identification using such multi-omics data (Chen *et al*. 2023, Hassan *et al*. 2025, Tan *et al*. 2025) to understand and treat human diseases. Unfortunately, the accuracy of those machine-learning models displays large variations even for the same clinical outcome due to many technical and confounding factors. These inconsistencies markedly disoriented our understanding of association, causation, and molecular biomarkers of the clinical outcomes. In this study, we propose a novel feature selection strategy that integrates species prevalence into standard importance metrics from machine learning models to enhance biomarker identification in both real and simulated microbiome datasets. Through a comprehensive series of simulations and analyses of real-world data, we demonstrate that incorporating prevalence information significantly improves the stability of selected features. Specifically, our novel approach reduces variability and improves reproducibility for microbiome-based biomarker identification.

Our results also reveal a strong and often overlooked association between taxon prevalence and feature selection frequency. In both synthetic and real microbiome datasets, more prevalent taxa were more likely to be selected by traditional random forest-based importance metrics. However, this implicit bias toward prevalent features can lead to instability and spurious biomarker identification, especially in sparse microbiome datasets where many taxa appear infrequently. By integrating scaled prevalence (e.g. prevalence^2.5^) into feature importance scores, we were able to improve the reproducibility of selected features across different sample sizes and simulation runs. Notably, this integrated approach yielded higher overlap of selected features and better classification performance across multiple evaluation metrics.

In applying our framework to real-world CRC datasets (Zeller *et al*. 2014, Baxter *et al*. 2016), we robustly identified well-established microbial genera such as *Ruminococcus* (Zhang *et al*. 2023), *Clostridium XVIII* (Xie *et al*. 2025), and *Faecalibacterium* (Dikeocha *et al*. 2022), as top-ranking features. These taxa have been repeatedly implicated in CRC pathogenesis and represent strong candidates for clinical biomarker development. Our method achieved robust selection of these taxa across different sample sizes and ranking strategies, suggesting improved robustness in the face of common challenges such as sample imbalance and noise. To account for the influence of prevalence on biomarker selection, we incorporated prevalence weighting to prioritize taxa consistently observed across samples. Low-prevalence taxa often show high variability and limited reproducibility across cohorts, increasing the risk of unstable or non-replicable findings. By emphasizing higher-prevalence features, our approach improves feature stability while maintaining predictive performance, as confirmed by external validation across independent CRC cohorts. However, our method prioritizes features based on prevalence, which may underrepresent rare but biologically important taxa.

Several alternative feature selection methods have been applied in microbiome data analysis, including LASSO-based approaches, Elastic Net, Relief-based algorithms, mRMR, and PreLect. Each offers distinct strengths but also faces limitations in microbial data. LASSO performs automatic variable selection by shrinking irrelevant coefficients to zero, thereby identifying relevant predictors. However, it may be unstable when features are highly correlated (Zou and Hastie 2005). Elastic Net on the other hand handles correlated variables better, but it still relies on linear modeling assumptions and does not explicitly address zero inflation (Zhang *et al*. 2020). Relief-based methods capture interactions and do not assume linearity; however, they are sensitive to noisy distance metrics (Robnik-Šikonja and Kononenko 2003). Similarly, mRMR avoids selecting redundant variables, but it can be sensitive to sample size and noise in sparse settings (Peng *et al*. 2005). PreLect is a more recent advancement that explicitly incorporates microbial prevalence into the feature selection process through a penalized regression framework. While PreLect directly incorporates prevalence and improves robustness under sparsity, it requires parameter tuning, which may increase computational complexity in large multi-cohort analyses (Chen *et al*. 2025). In contrast, ParSlet provides a simple adjustment to standard feature importance scores that directly includes prevalence information. Specifically, we noted that ParSlet and PreLect show complementary performance under certain settings in both simulation and real data. In practice, users may consider using both methods and focusing on the overlapping microbial features, which are more likely to be robust and reliable signals.

Beyond microbiome data, our prevalence-aware feature selection framework has potential applications in single-cell omics, where data sparsity, dropout events, and prevalence bias are even more pronounced. In single-cell RNA sequencing, for instance, many genes are detected at low prevalence across cells, making biomarker discovery highly unstable with traditional importance metrics (Andrews and Hemberg 2019). Incorporating cell-level prevalence into feature importance scores could enhance the reproducibility of cell-type-specific markers and improve classification of disease-associated subpopulations. Similarly, in single-cell multi-omics, integrating prevalence information may help stabilize feature ranking across heterogeneous cells and replicates, thereby improving the robustness of biomarker discovery.

Previous studies have also found some other laws, e.g. data separation law in deep learning, implying that the neural network makes equal progress in reducing data separation fuzziness (in log scale) in classification problems over each layer on the training data (He and Su 2023). Combining those training laws with our prediction law can help us understand the origin of prediction law, which warrants further study in the future.

We acknowledge several limitations of our study. First, the use of simulated data provides a controlled framework with known ground truth, which allows us to manipulate effect sizes, prevalence levels, and sample sizes with precision. However, although the simulated data were parameterized using real human microbiome profiles, simulation-based findings may not fully capture the complex and dynamic nature of real-world microbial ecosystems. Future work should therefore focus on developing more biologically informed simulation frameworks capable of modeling complex, non-linear ecological interactions and functional dynamics. Second, evaluating stability using repeated subsampling from the same dataset may introduce dependence due to overlapping training-test splits derived from a single underlying dataset. Third, because the proposed framework prioritizes features based on prevalence, it may underrepresent rare taxa that are biologically important but present in only a small subset of samples. Finally, while we identified consistent colorectal cancer (CRC) associated microbial biomarkers across datasets, these findings are based on statistical associations. Our study does not establish causal relationships between specific taxa and disease development, and further experimental validation will be necessary in future studies.

In conclusion, we developed ParSlet, a user-friendly computational framework for identifying reproducible microbiome features across datasets, and evaluated its performance using both simulated and real-world microbiome data. Together, these findings support ParSlet as a practical tool for microbiome feature discovery and provide a foundation for future development of microbiome-based therapeutics.

## Supplementary Material

btag371_Supplementary_Data

## Data Availability

All the real-world microbiome data used in this article come from publicly available sources (https://figshare.com/articles/dataset/16S_rRNA_Microbiome_Datasets/14531724). The codes for data simulation, statistical analyses, and visualization are available in the GitHub repository (https://github.com/KelabatOSU/Feature_selection).

## References

[btag371-B1] Andrews TS , Hemberg M. M3Drop: dropout-based feature selection for scRNASeq. Bioinformatics 2019;35:2865–7. 10.1093/bioinformatics/bty1044 30590489 PMC6691329

[btag371-B2] Babu M , Snyder M. Multi-omics profiling for health. Mol Cell Proteomics MCP 2023;22:100561. 10.1016/j.mcpro.2023.100561 37119971 PMC10220275

[btag371-B3] Barcik W , Boutin RCT, Sokolowska M et al The role of lung and gut microbiota in the pathology of asthma. Immunity 2020;52:241–55. 10.1016/j.immuni.2020.01.007 32075727 PMC7128389

[btag371-B4] Baxter NT , Ruffin MT, Rogers MAM et al Microbiota-based model improves the sensitivity of fecal immunochemical test for detecting colonic lesions. Genome Med 2016;8:37. 10.1186/s13073-016-0290-3 27056827 PMC4823848

[btag371-B5] Britton RA , Young VB. Role of the intestinal microbiota in resistance to colonization by clostridium difficile. Gastroenterology 2014;146:1547–53. 10.1053/j.gastro.2014.01.059 24503131 PMC3995857

[btag371-B6] Chen Y , Wen Y, Xie C et al MOCSS: Multi-omics data clustering and cancer subtyping via shared and specific representation learning. iScience 2023;26:107378. 10.1016/j.isci.2023.107378 37559907 PMC10407241

[btag371-B7] Chen Y-C , Su Y-Y, Chu T-Y et al PreLect: Prevalence leveraged consistent feature selection decodes microbial signatures across cohorts. NPJ Biofilms Microbiomes 2025;11:3. 10.1038/s41522-024-00598-2 39753565 PMC11698977

[btag371-B8] Cox DR. The regression analysis of binary sequences. J R Stat Soc Ser B (Methodological) 1958;20:215–32. 10.1111/j.2517-6161.1958.tb00292.x

[btag371-B9] Dai D , Zhu J, Sun C et al GMrepo v2: A curated human gut microbiome database with special focus on disease markers and cross-dataset comparison. Nucleic Acids Res 2022;50:D777–84. 10.1093/nar/gkab1019 34788838 PMC8728112

[btag371-B10] Dikeocha IJ , Al-Kabsi AM, Chiu H-T et al *Faecalibacterium prausnitzii* ameliorates colorectal tumorigenesis and suppresses proliferation of HCT116 colorectal cancer cells. Biomedicines 2022;10:1128. 10.3390/biomedicines10051128 35625865 PMC9138996

[btag371-B11] Duvallet C , Gibbons SM, Gurry T et al Meta-analysis of gut microbiome studies identifies disease-specific and shared responses. Nat Commun 2017;8:1784. 10.1038/s41467-017-01973-8 29209090 PMC5716994

[btag371-B12] Franzosa EA , Sirota-Madi A, Avila-Pacheco J et al Gut microbiome structure and metabolic activity in inflammatory bowel disease. Nat Microbiol 2019;4:293–305. 10.1038/s41564-018-0306-4 30531976 PMC6342642

[btag371-B13] Fromentin S , Forslund SK, Chechi K et al Microbiome and metabolome features of the cardiometabolic disease spectrum. Nat Med 2022;28:303–14. 10.1038/s41591-022-01688-4 35177860 PMC8863577

[btag371-B14] Gonzales-Luna AJ , Carlson TJ, Garey KW. Gut microbiota changes associated with Clostridioides difficile infection and its various treatment strategies. Gut Microbes 2023;15:2223345. 10.1080/19490976.2023.2223345 37318134 PMC10274544

[btag371-B15] Hasin Y , Seldin M, Lusis A. Multi-omics approaches to disease. Genome Biol 2017;18:83. 10.1186/s13059-017-1215-1 28476144 PMC5418815

[btag371-B16] Hassan AM , Naeem SM, Eldosoky MAA et al Multi-omics-based machine learning for the subtype classification of breast cancer. Arabian J Sci Eng 2025;50:1339–52. 10.1007/s13369-024-09341-7

[btag371-B17] Hastie T , Tibshirani R. Generalized additive models. Stat Sci 1986;1:297–310. 10.1214/ss/1177013604 8548102

[btag371-B18] He H , Su WJ. A law of data separation in deep learning. Proc Natl Acad Sci USA 2023;120:e2221704120. 10.1073/pnas.2221704120 37639604 PMC10483613

[btag371-B19] John Lu ZQ. The elements of statistical learning: data mining, inference, and prediction. J R Stat Soc Ser A Stat Soc 2010;173:693–4. 10.1111/j.1467-985X.2010.00646_6.x

[btag371-B20] Karczewski KJ , Snyder MP. Integrative omics for health and disease. Nat Rev Genet 2018;19:299–310. 10.1038/nrg.2018.4 29479082 PMC5990367

[btag371-B21] Karwowska Z , Aasmets O, Metspalu M et al; Estonian Biobank Research Team. Effects of data transformation and model selection on feature importance in microbiome classification data. Microbiome 2025;13:2. 10.1186/s40168-024-01996-6 39754220 PMC11699698

[btag371-B22] Kira K , Rendell LA. A practical approach to feature selection. In: Machine Learning Proceedings 1992, *Aberdeen, Scotland, UK*, 1-3 July 1992. p. 249–56. San Francisco, CA, USA: Morgan Kaufmann. 1992. 10.1016/B978-1-55860-247-2.50037-1

[btag371-B23] Kolisnik T , Sulit AK, Schmeier S et al Identifying important microbial and genomic biomarkers for differentiating right- versus left-sided colorectal cancer using random forest models. BMC Cancer 2023;23:647. 10.1186/s12885-023-10848-9 37434131 PMC10337110

[btag371-B24] Lee SJ , Rho M. Multimodal deep learning applied to classify healthy and disease states of human microbiome. Sci Rep 2022;12:824. 10.1038/s41598-022-04773-3 35039534 PMC8763943

[btag371-B25] Liang W , Yang Y, Gong S et al Airway dysbiosis accelerates lung function decline in chronic obstructive pulmonary disease. Cell Host Microbe 2023;31:1054–70.e9. 10.1016/j.chom.2023.04.018 37207649

[btag371-B26] Liaw A, Wiener M. Classification and regression by randomforest. *R News.* 2002;**2**:18–22. http://CRAN.R-project.org/doc/Rnews/ (18 August 2025, date last accessed).

[btag371-B27] Ma S , Ren B, Mallick H et al A statistical model for describing and simulating microbial community profiles. PLoS Comput Biol 2021;17:e1008913. 10.1371/journal.pcbi.1008913 34516542 PMC8491899

[btag371-B28] Marcos-Zambrano LJ , Karaduzovic-Hadziabdic K, Loncar Turukalo T *et al.* Applications of machine learning in human microbiome studies: A review on feature selection, biomarker identification, disease prediction and treatment. *Front Microbiol* 2021;**12**:634511. 10.3389/fmicb.2021.634511 PMC7962872 33737920

[btag371-B29] Martín R , Rios-Covian D, Huillet E et al Faecalibacterium: A bacterial genus with promising human health applications. FEMS Microbiol Rev 2023;47:fuad039. 10.1093/femsre/fuad039 37451743 PMC10410495

[btag371-B30] Nearing JT , Douglas GM, Hayes MG et al Microbiome differential abundance methods produce different results across 38 datasets. Nat Commun 2022;13:342. 10.1038/s41467-022-28034-z 35039521 PMC8763921

[btag371-B31] Ogunrinola GA , Oyewale JO, Oshamika OO et al The human microbiome and its impacts on health. Int J Microbiol 2020;2020:8045646. 10.1155/2020/8045646 32612660 PMC7306068

[btag371-B32] Ott SJ, Musfeldt M, Wenderoth DF *et al*. Reduction in diversity of the colonic mucosa associated bacterial microflora in patients with active inflammatory bowel disease. *Gut* 2004;**53**:685–93. 10.1136/gut.2003.025403 PMC1774050 15082587

[btag371-B33] Pasolli E , Schiffer L, Manghi P et al Accessible, curated metagenomic data through ExperimentHub. Nat Methods 2017;14:1023–4. 10.1038/nmeth.4468 29088129 PMC5862039

[btag371-B34] Peng H , Long F, Ding C. Feature selection based on mutual information criteria of max-dependency, max-relevance, and min-redundancy. IEEE Trans Pattern Anal Mach Intell 2005;27:1226–38. 10.1109/TPAMI.2005.159 16119262

[btag371-B35] Piccolo SR , Mecham A, Golightly NP et al The ability to classify patients based on gene-expression data varies by algorithm and performance metric. PLOS Comput Biol 2022;18:e1009926. 10.1371/journal.pcbi.1009926 35275931 PMC8942277

[btag371-B36] Reinsch CH. Smoothing by spline functions. Numerische Mathematik 1967;10:177–83. 10.1007/BF02162161

[btag371-B37] Robnik-Šikonja M , Kononenko I. Theoretical and Empirical Analysis of ReliefF and RReliefF. Mach Learn 2003;53:23–69. 10.1023/A:1025667309714

[btag371-B38] Sun W , Zhang Y, Guo R et al A population-scale analysis of 36 gut microbiome studies reveals universal species signatures for common diseases. NPJ Biofilms Microbiomes 2024;10:96. 10.1038/s41522-024-00567-9 39349486 PMC11442664

[btag371-B39] Tan CY , Ong HF, Lim CH et al Amogel: A multi-omics classification framework using associative graph neural networks with prior knowledge for biomarker identification. BMC Bioinformatics 2025;26:94. 10.1186/s12859-025-06111-6 40155814 PMC11954243

[btag371-B40] Tibshirani R. Regression shrinkage and selection via the Lasso. J R Stat Soc Ser B (Methodological) 1996;58:267–88. 10.1111/j.2517-6161.1996.tb02080.x

[btag371-B41] Wang X-W , Wang T, Schaub DP et al Benchmarking omics-based prediction of asthma development in children. Respiratory Res 2023;24:63. 10.1186/s12931-023-02368-8 PMC9969629 36842969

[btag371-B42] Wang Y , Bhattacharya T, Jiang Y et al A novel deep learning method for predictive modeling of microbiome data. Brief Bioinf 2021;22:bbaa073. 10.1093/bib/bbaa073 PMC13017428 32406914

[btag371-B43] Wu S , Rhee K-J, Albesiano E et al A human colonic commensal promotes colon tumorigenesis via activation of T helper type 17 T cell responses. Nat Med 2009;15:1016–22. 10.1038/nm.2015 19701202 PMC3034219

[btag371-B44] Xie M, Yuan K, Zhang Y *et al*. Tumor-resident probiotic *Clostridium butyricum* improves a PD-1 efficacy in colorectal cancer models by inhibiting IL-6-mediated immunosuppression. *Cancer Cell* 2025;**43**:1885–901.e10. 10.1016/j.ccell.2025.07.012 40780216

[btag371-B45] Yang H, Wu J, Huang X *et al. ABO* genotype alters the gut microbiota by regulating GalNAc levels in pigs. *Nature* 2022;**606**:358–367. 10.1038/s41586-022-04769-z PMC9157047 35477154

[btag371-B46] Zeller G , Tap J, Voigt AY et al Potential of fecal microbiota for early‐stage detection of colorectal cancer. Mol Syst Biol 2014;10:766. 10.15252/msb.20145645 25432777 PMC4299606

[btag371-B47] Zhang X , Guo B, Yi N. Zero-Inflated gaussian mixed models for analyzing longitudinal microbiome data. PLoS One 2020;15:e0242073. 10.1371/journal.pone.0242073 33166356 PMC7652264

[btag371-B48] Zhang X , Yu D, Wu D et al Tissue-resident Lachnospiraceae family bacteria protect against colorectal carcinogenesis by promoting tumor immune surveillance. Cell Host Microbe 2023;31:418–32.e8. 10.1016/j.chom.2023.01.013 36893736

[btag371-B49] Zou H , Hastie T. Regularization and variable selection via the elastic net. J R Stat Soc Ser B Stat Methodol 2005;67:301–20. 10.1111/j.1467-9868.2005.00503.x

