# Supplementary material for: Prevalence aware feature selection improves biomarker identification in microbiome studies
Source: Bioinformatics. 2026 Jun 24;42(7):btag371. doi: 10.1093/bioinformatics/btag371 (PMC13326745; doi:10.1093/bioinformatics/btag371)
Supplement: btag371_Supplementary_Data [file btag371_supplementary_data.docx]

**Supplementary Information**

**
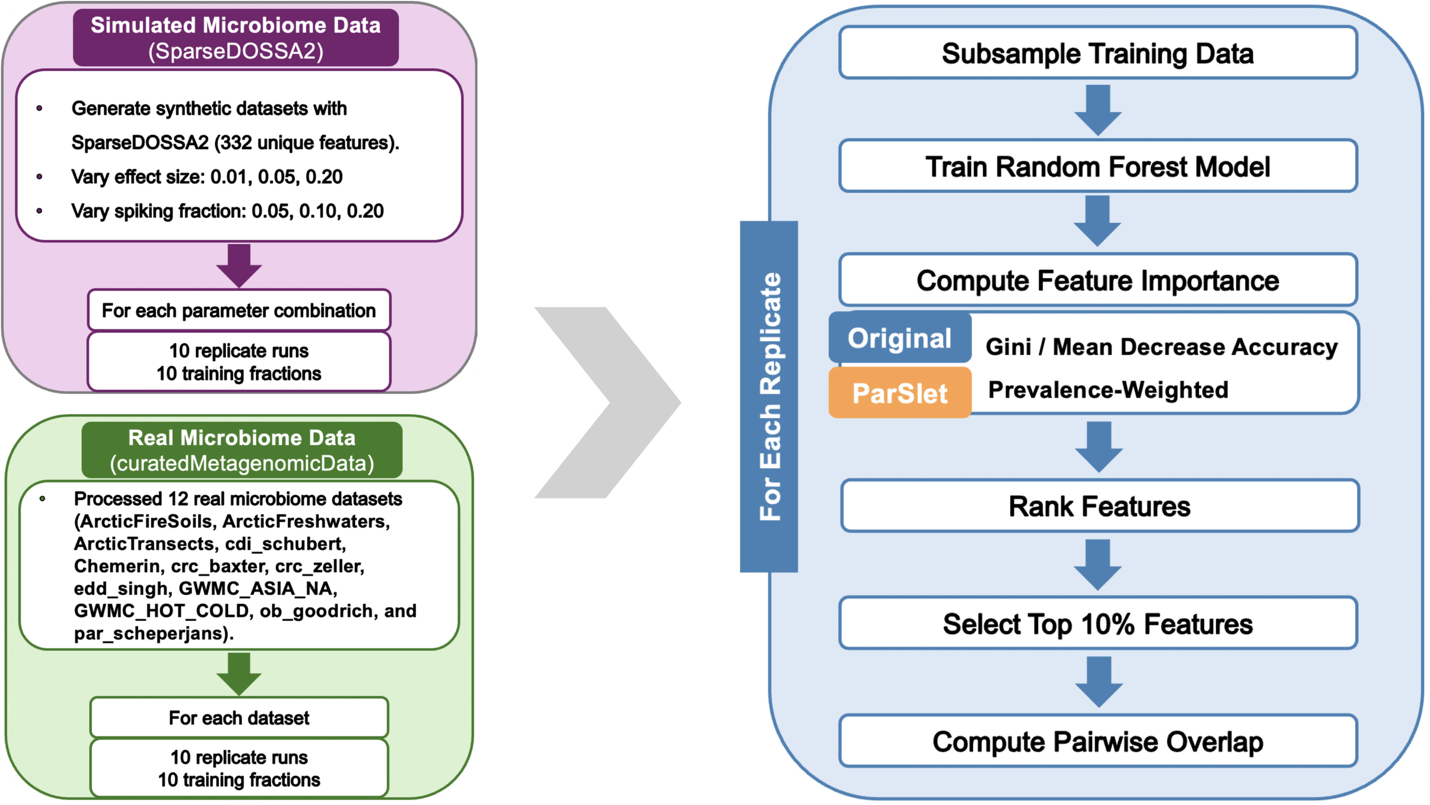
**

**Figure S1. Overview of the feature selection and comparison framework for simulated and real microbiome data.** Simulated microbiome datasets (purple; generated using SparseDOSSA2) and real microbiome datasets (green; curatedMetagenomicData) are processed through a unified analysis pipeline. For each dataset or parameter combination, 10 replicates and 10 training fractions are generated. Training data are subsampled to compute feature importance using Random Forest models. Feature selection methods are compared under identical settings, including the original importance measures (Gini and mean decrease in accuracy; blue), the ParSlet approach (orange), and existing methods (e.g., LASSO, Elastic Net, Relief, mRMR, and PreLect). Features are ranked and the top 10% are selected. Stability is evaluated by computing pairwise overlap of selected features across training fractions and methods.

**
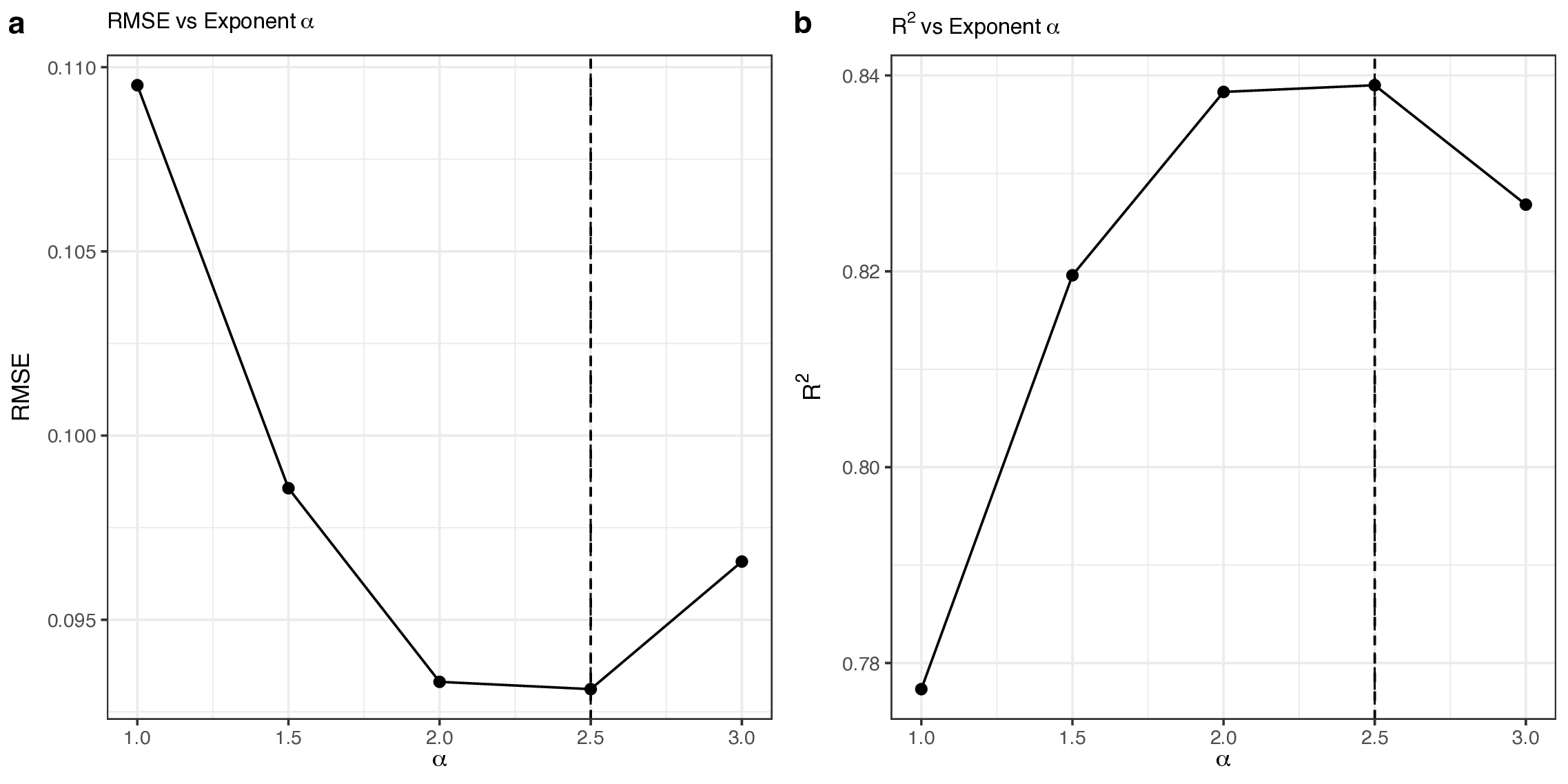
**

**Figure S2. Selection of the prevalence scaling exponent α based on model performance.** (a) Root mean squared error (RMSE) and (b) coefficient of determination (R²) as functions of the exponent α used in the power-law transformation of feature prevalence. For each candidate value of α (1.0-3.0), a regression model was fitted between transformed prevalence and feature selection frequency across repeated simulation runs. The dashed vertical line indicates α = 2.5, which yields the lowest RMSE and the highest and near-highest R² across simulation settings.

**
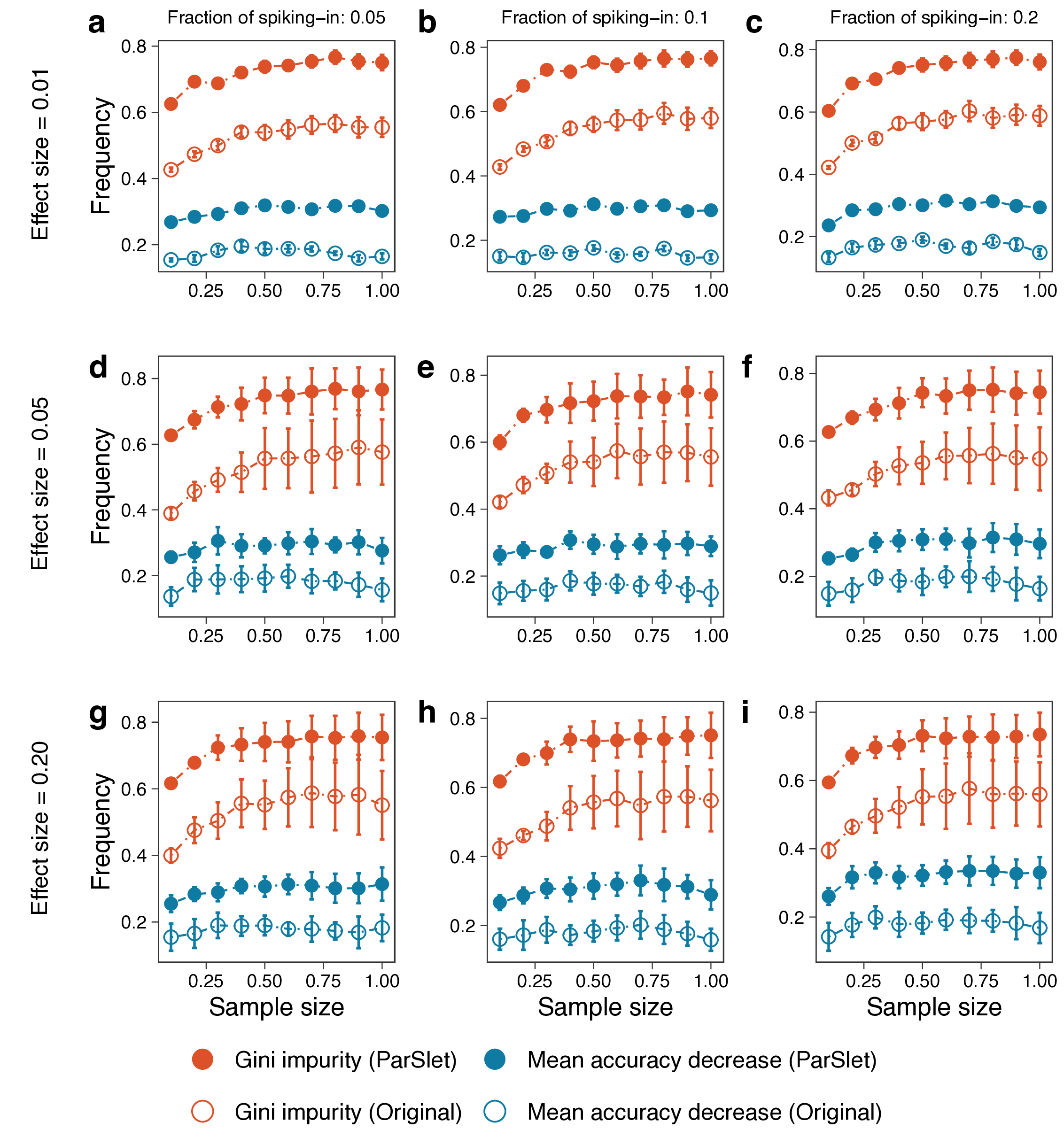
**

**Figure S3.** **Mean overlap of top 10% selected features across normalized sample sizes and spiking-in fractions using ParSlet.** The average pairwise overlap of the top 10% most important features is shown across normalized sample sizes ($f_{S}$​) under varying fractions of spiked-in taxa ($f_{D}=0.05, 0.1, 0.2$; panels a-i). Feature importance was ranked using either Gini impurity or Mean Decrease Accuracy, with or without prevalence integration (importance $\times$ richness^2.5^). ParSlet consistently show higher overlap than unadjusted rankings, suggesting improved feature selection stability across runs.

**
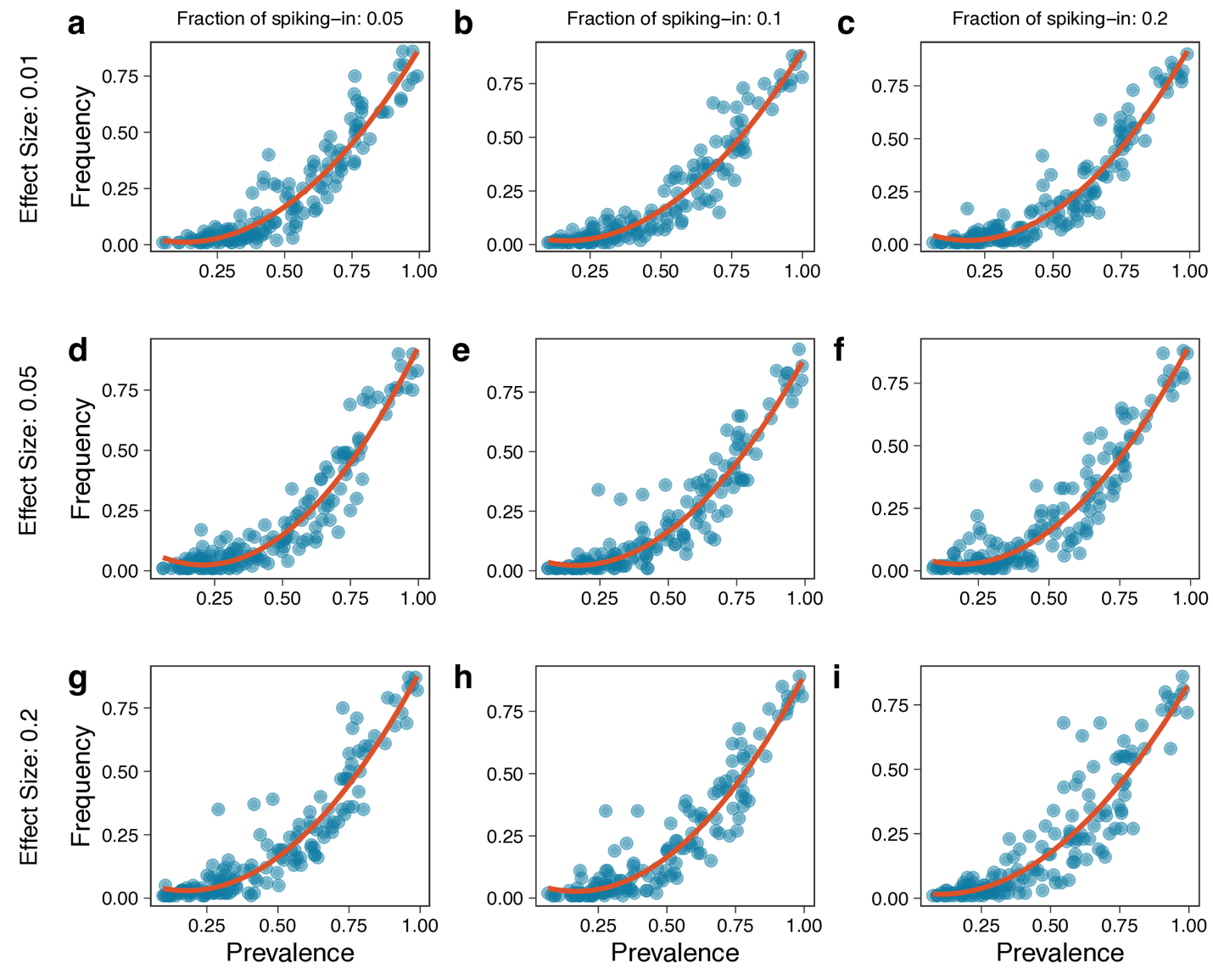
**

**Figure S4.** **Frequency of a feature’s inclusion among the top 10% most important features versus its prevalence using polynomial regression.** Each point represents a taxon, showing its average prevalence across simulated samples (x-axis) and the frequency with which it was selected among the top 10% most important features across all simulation runs (y-axis). Data are shown for simulated microbiome datasets under varying fractions of spiked-in taxa ($f_{D}=0.05, 0.1, 0.2$). A nonlinear positive association is observed across simulation settings, indicating that features with higher prevalence tend to be selected more frequently.

**
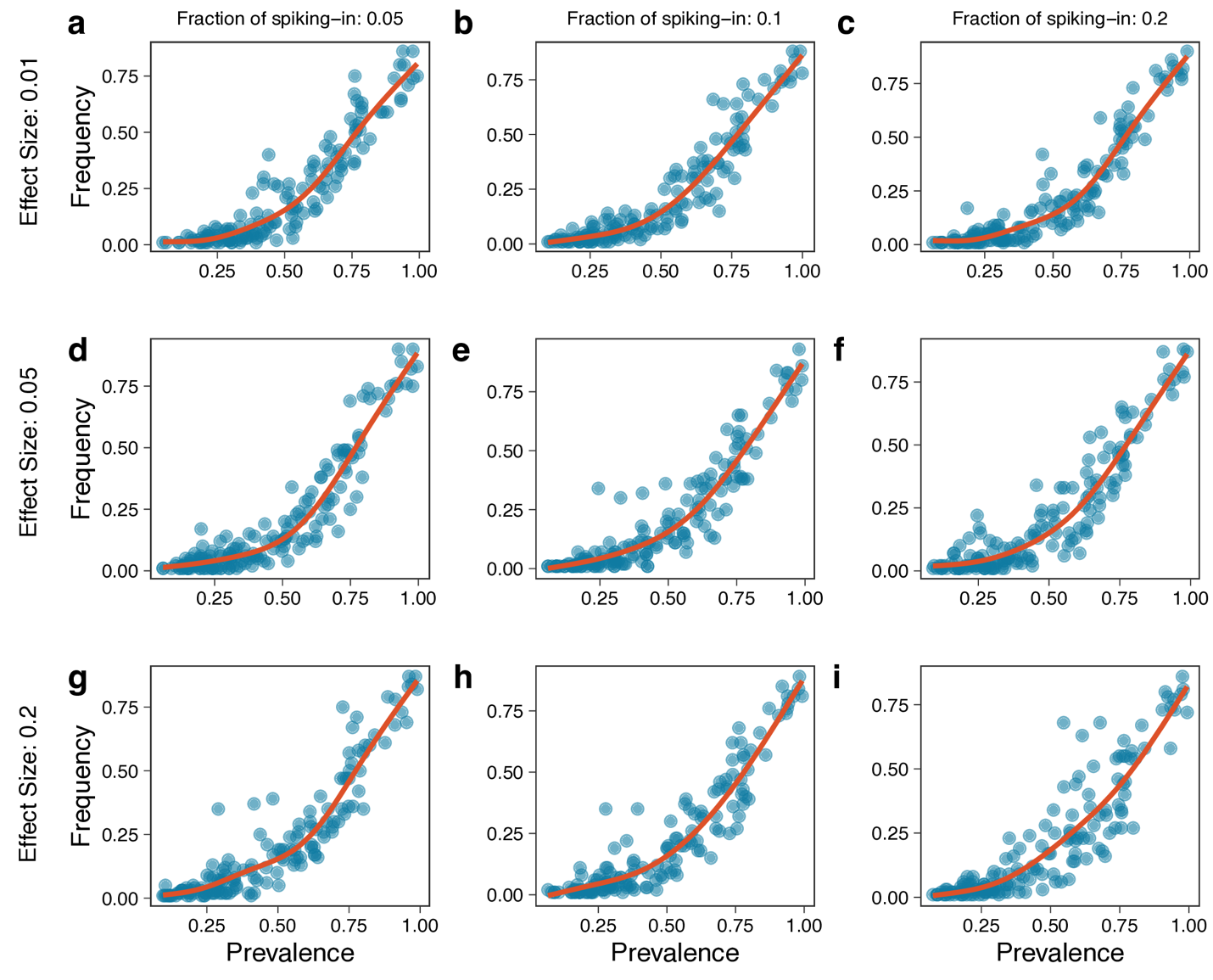
**

**Figure S5.** **Frequency of a feature’s inclusion among the top 10% most important features versus its prevalence using generalized additive models (GAMs).** Each point represents a taxon, showing its average prevalence across simulated samples (x-axis) and the frequency with which it was selected among the top 10% most important features across all simulation runs (y-axis). Data are shown for simulated microbiome datasets under varying fractions of spiked-in taxa ($f_{D}=0.05, 0.1, 0.2$). A nonlinear positive association is observed across simulation settings, indicating that features with higher prevalence tend to be selected more frequently.

**
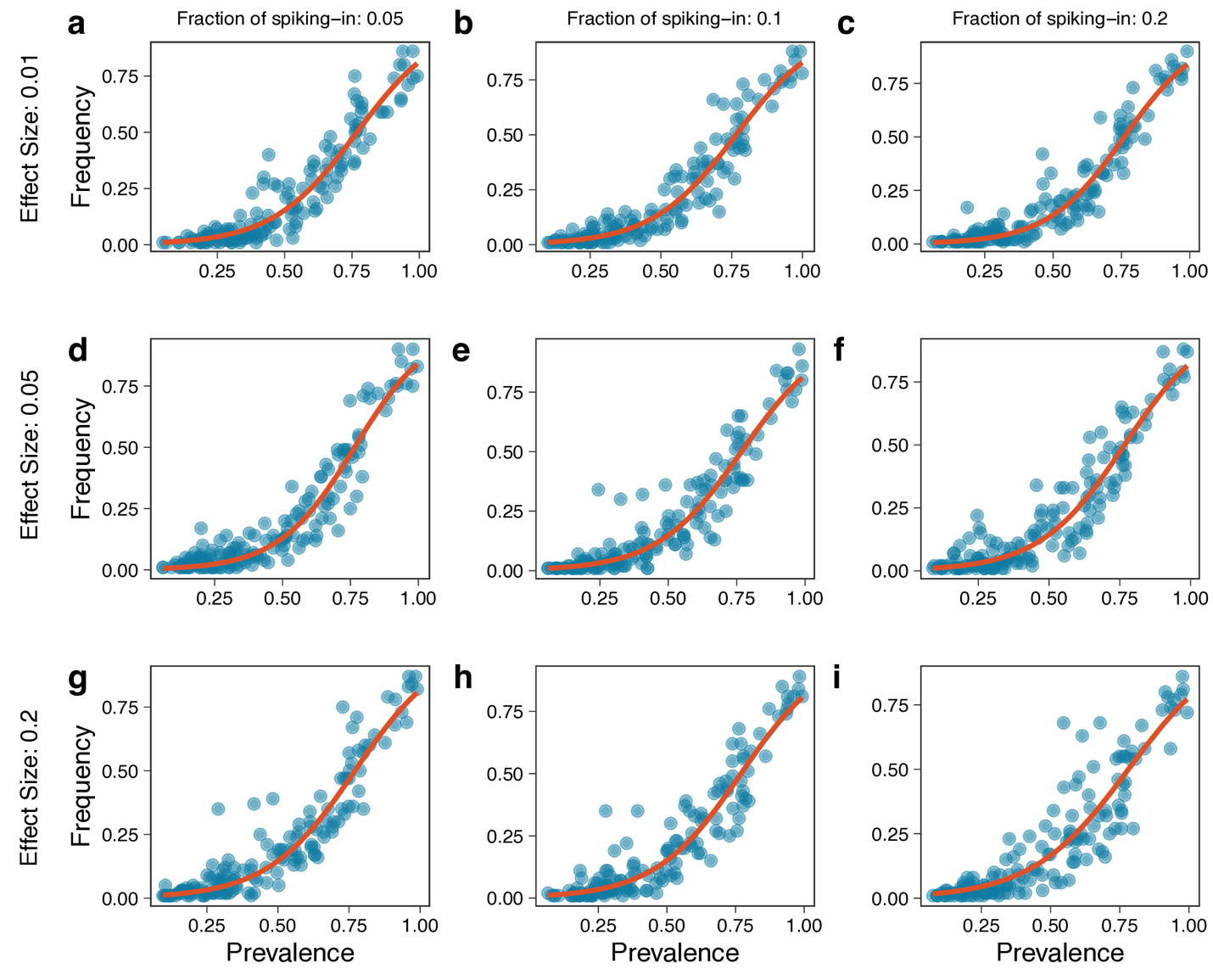
**

**Figure S6.** **Frequency of a feature’s inclusion among the top 10% most important features versus its prevalence using logistic curves.** Each point represents a taxon, showing its average prevalence across simulated samples (x-axis) and the frequency with which it was selected among the top 10% most important features across all simulation runs (y-axis). Data are shown for simulated microbiome datasets under varying fractions of spiked-in taxa ($f_{D}=0.05, 0.1, 0.2$). A nonlinear positive association is observed across simulation settings, indicating that features with higher prevalence tend to be selected more frequently.

**
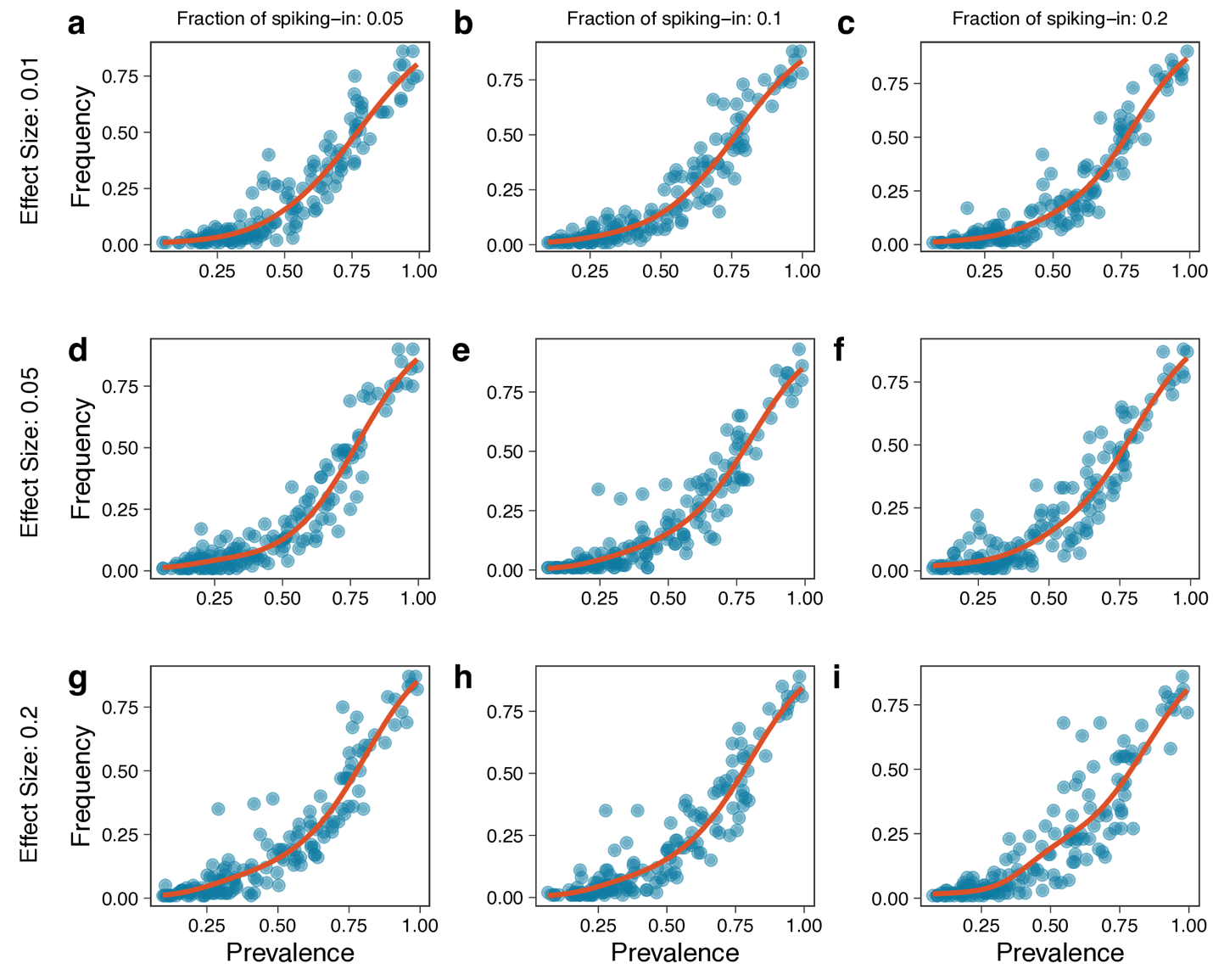
**

**Figure S7.** **Frequency of a feature’s inclusion among the top 10% most important features versus its prevalence using spline-based regression.** Each point represents a taxon, showing its average prevalence across simulated samples (x-axis) and the frequency with which it was selected among the top 10% most important features across all simulation runs (y-axis). Data are shown for simulated microbiome datasets under varying fractions of spiked-in taxa ($f_{D}=0.05, 0.1, 0.2$). A nonlinear positive association is observed across simulation settings, indicating that features with higher prevalence tend to be selected more frequently.

**
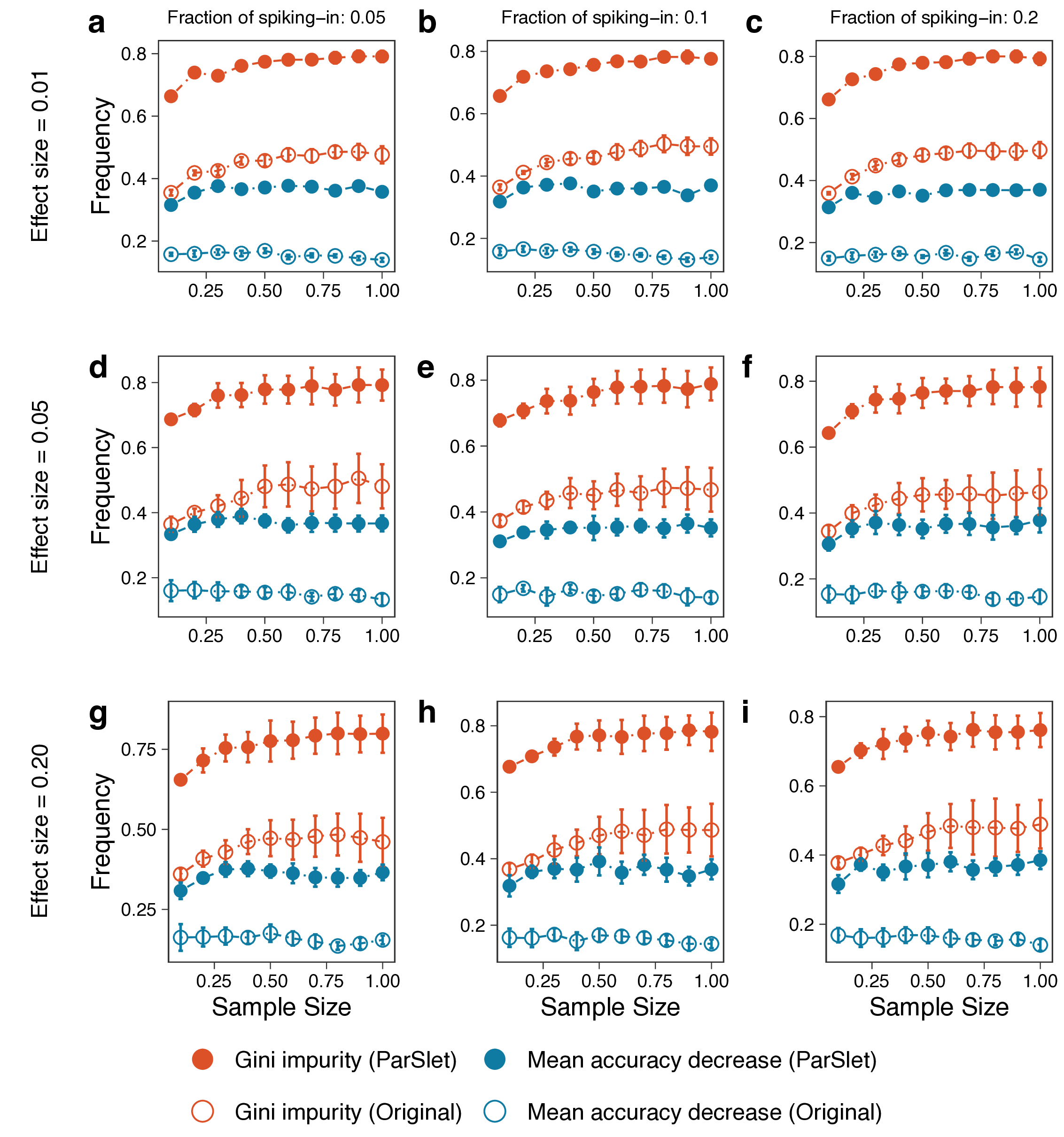
**

**Figure S8. Mean overlap of top 10% selected features across normalized sample sizes and spiking-in fractions using ParSlet (with ntree=100).** The average pairwise overlap of the top 10% most important features is shown across normalized sample sizes ($f_{S}$​) under varying fractions of spiked-in taxa ($f_{D}=0.05, 0.1, 0.2$; panels a-i). Feature importance was ranked using either Gini impurity or Mean Decrease Accuracy, with or without prevalence integration (importance x richness^2.5^). ParSlet rankings consistently show higher overlap than unadjusted rankings, suggesting improved feature selection stability across runs.

**
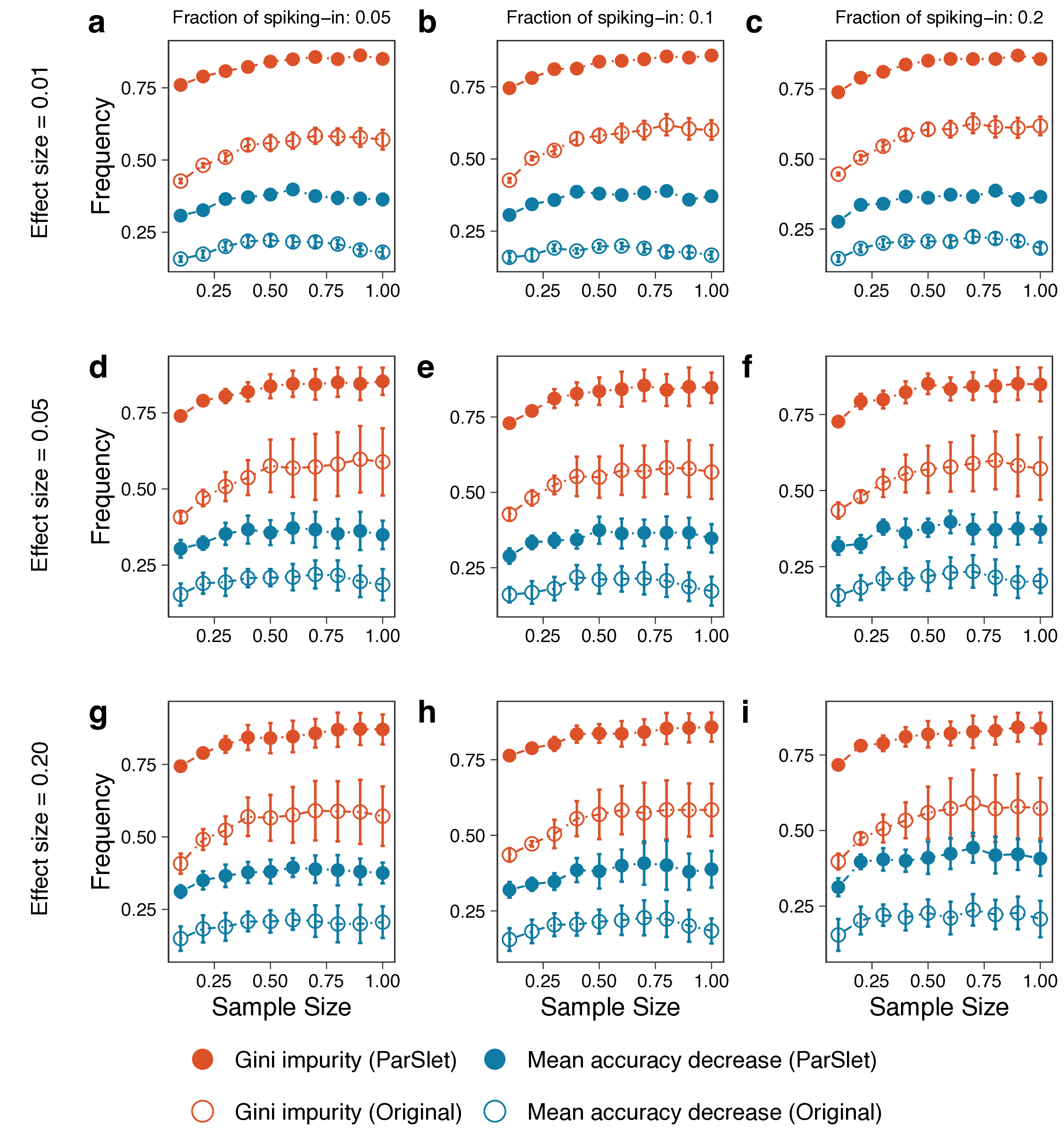
**

**Figure S9. Mean overlap of top 10% selected features across normalized sample sizes and spiking-in fractions using ParSlet (with ntree=1000).** The average pairwise overlap of the top 10% most important features is shown across normalized sample sizes ($f_{S}$​) under varying fractions of spiked-in taxa ($f_{D}=0.05, 0.1, 0.2$; panels a-i). Feature importance was ranked using either Gini impurity or Mean Decrease Accuracy, with or without prevalence integration (importance x richness^2.5^). ParSlet rankings consistently show higher overlap than unadjusted rankings, suggesting improved feature selection stability across runs.

**
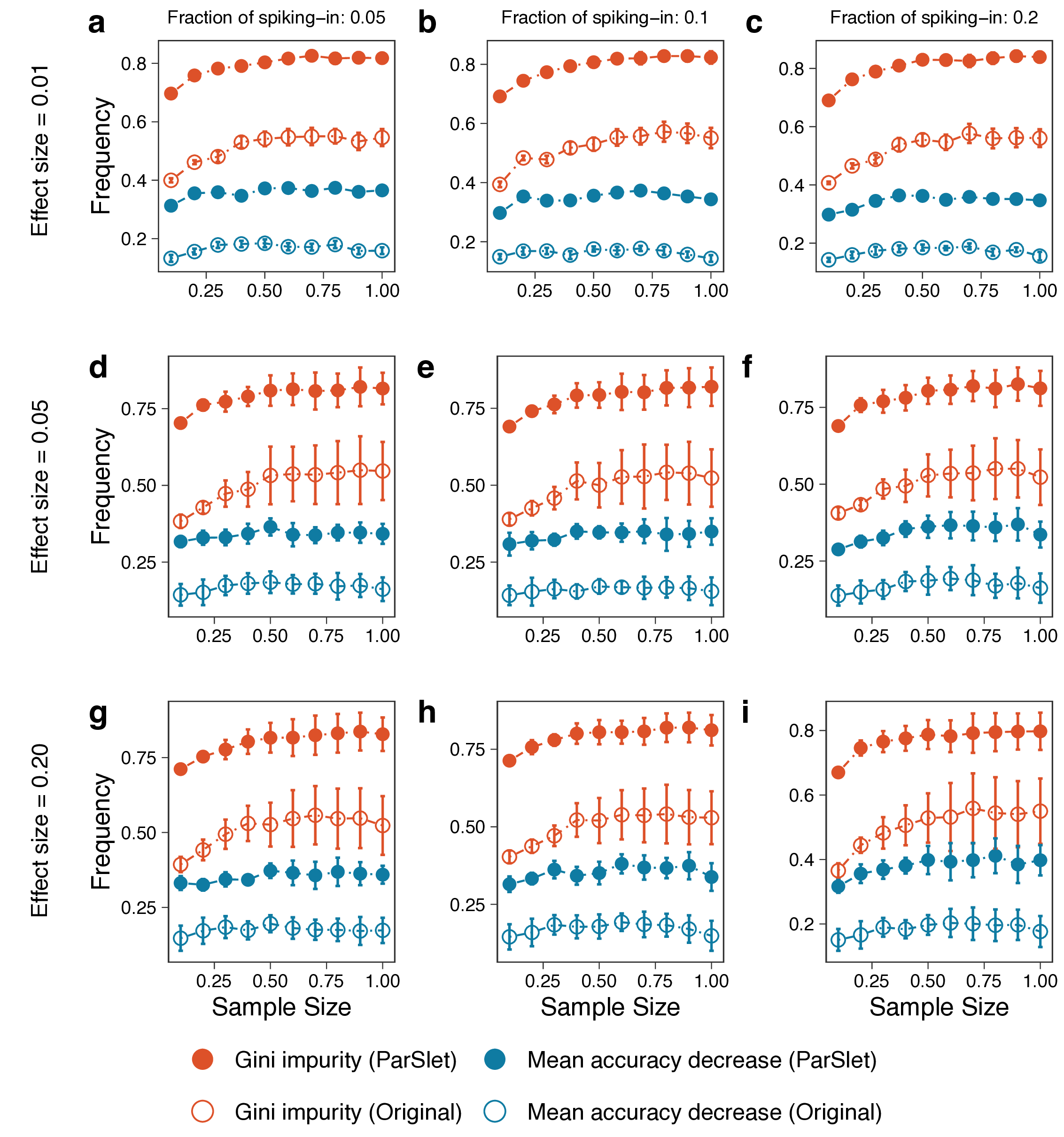
**

**Figure S10. Mean overlap of top 10% selected features across normalized sample sizes and spiking-in fractions using ParSlet (with mtry=p/10).** The average pairwise overlap of the top 10% most important features is shown across normalized sample sizes ($f_{S}$​) under varying fractions of spiked-in taxa ($f_{D}=0.05, 0.1, 0.2$; panels a-i). Feature importance was ranked using either Gini impurity or Mean Decrease Accuracy, with or without prevalence integration (importance x richness^2.5^). ParSlet rankings consistently show higher overlap than unadjusted rankings, suggesting improved feature selection stability across runs.

**
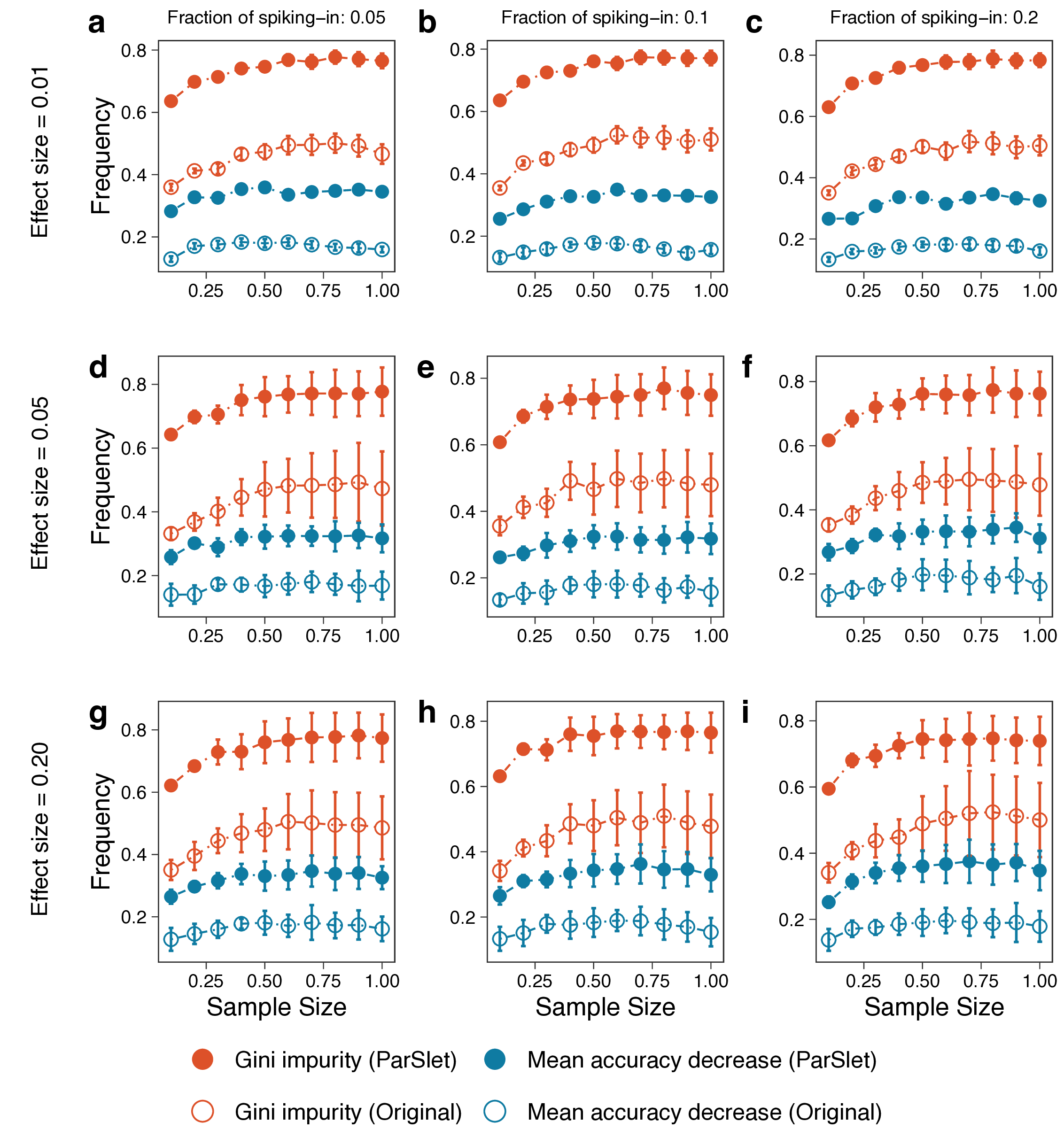
**

**Figure S11. Mean overlap of top 10% selected features across normalized sample sizes and spiking-in fractions using ParSlet (with mtry=p/2).** The average pairwise overlap of the top 10% most important features is shown across normalized sample sizes ($f_{S}$​) under varying fractions of spiked-in taxa ($f_{D}=0.05, 0.1, 0.2$; panels a-i). Feature importance was ranked using either Gini impurity or Mean Decrease Accuracy, with or without prevalence integration (importance x richness^2.5^). ParSlet rankings consistently show higher overlap than unadjusted rankings, suggesting improved feature selection stability across runs.

**
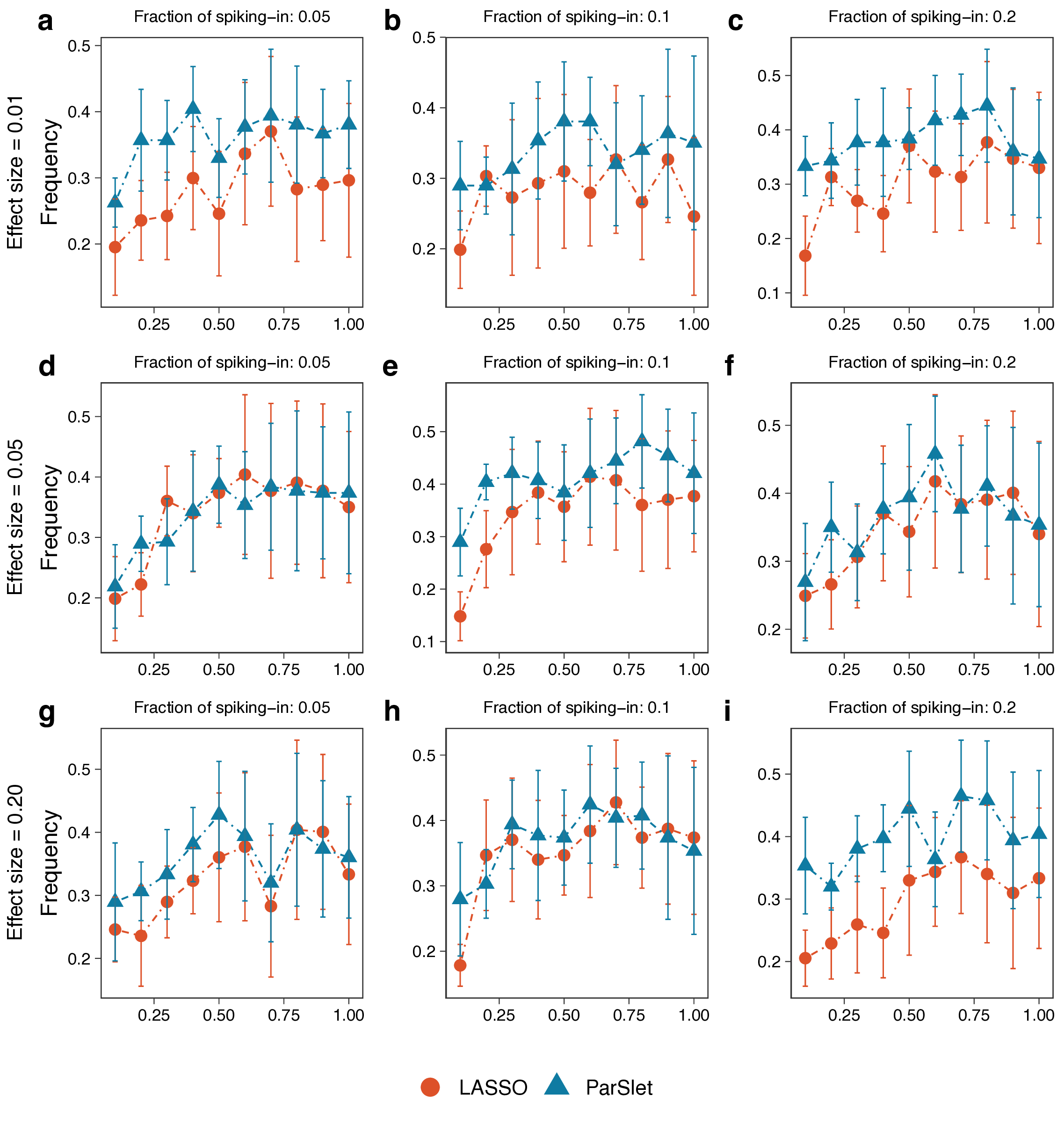
**

**Figure S12. Stability of feature selection under different effect sizes and spiking fractions using LASSO and ParSlet.** Each row corresponds to a different effect size (0.01, 0.05, 0.20). Each column corresponds to a different fraction of spiked-in features (0.05, 0.10, 0.20). The x-axis shows the fraction of samples used for training, and the y-axis shows the mean overlap of the top 10% ranked features between pairs of subsampled datasets. Red circles represent the standard LASSO ranking, while blue triangles represent the ParSlet ranking, in which feature scores are multiplied by prevalence raised to the power of 2.5.

**
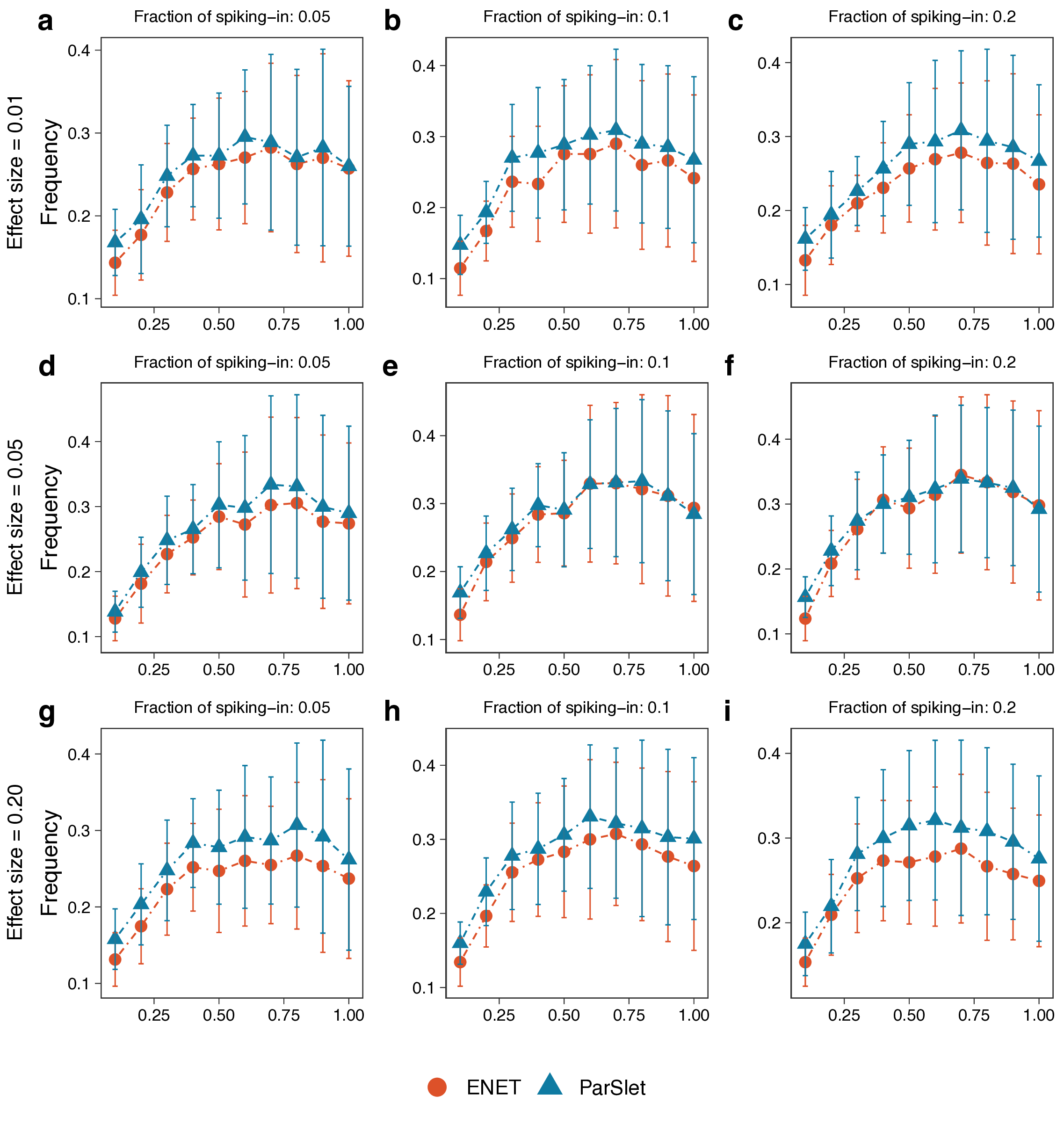
**

**Figure S13. Stability of feature selection under different effect sizes and spiking fractions using Elastic Net and ParSlet.** Each row corresponds to a different effect size (0.01, 0.05, 0.20). Each column corresponds to a different fraction of spiked-in features (0.05, 0.10, 0.20). The x-axis shows the fraction of samples used for training, and the y-axis shows the mean overlap of the top 10% ranked features between pairs of subsampled datasets. Red circles represent the standard Elastic Net ranking, while blue triangles represent the ParSlet ranking, in which feature scores are multiplied by prevalence raised to the power of 2.5.

**
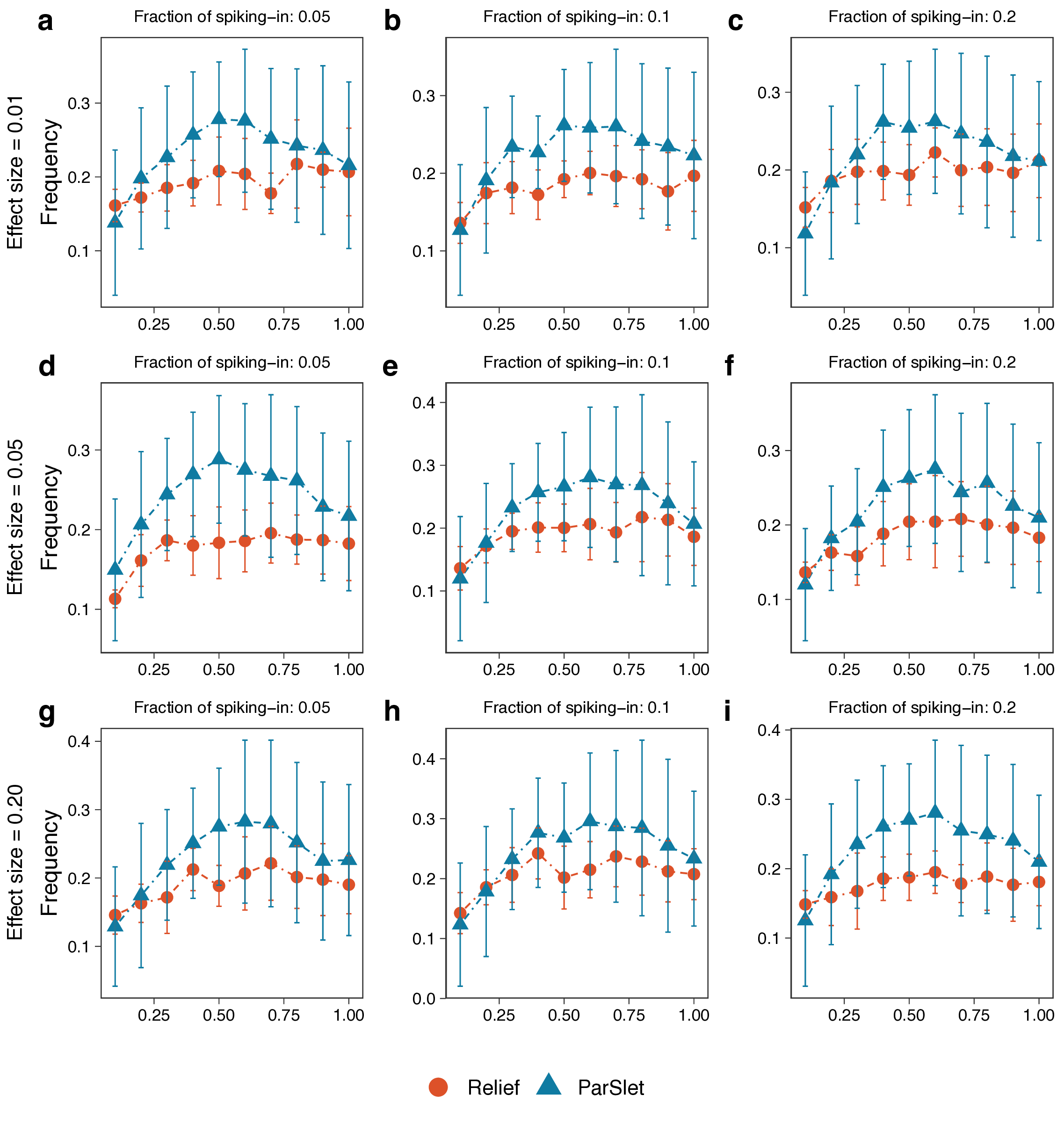
**

**Figure S14. Stability of feature selection under different effect sizes and spiking fractions using Relief and ParSlet.** Each row corresponds to a different effect size (0.01, 0.05, 0.20). Each column corresponds to a different fraction of spiked-in features (0.05, 0.10, 0.20). The x-axis shows the fraction of samples used for training, and the y-axis shows the mean overlap of the top 10% ranked features between pairs of subsampled datasets. Red circles represent the standard Relief ranking, while blue triangles represent the ParSlet ranking, in which feature scores are multiplied by prevalence raised to the power of 2.5.

**
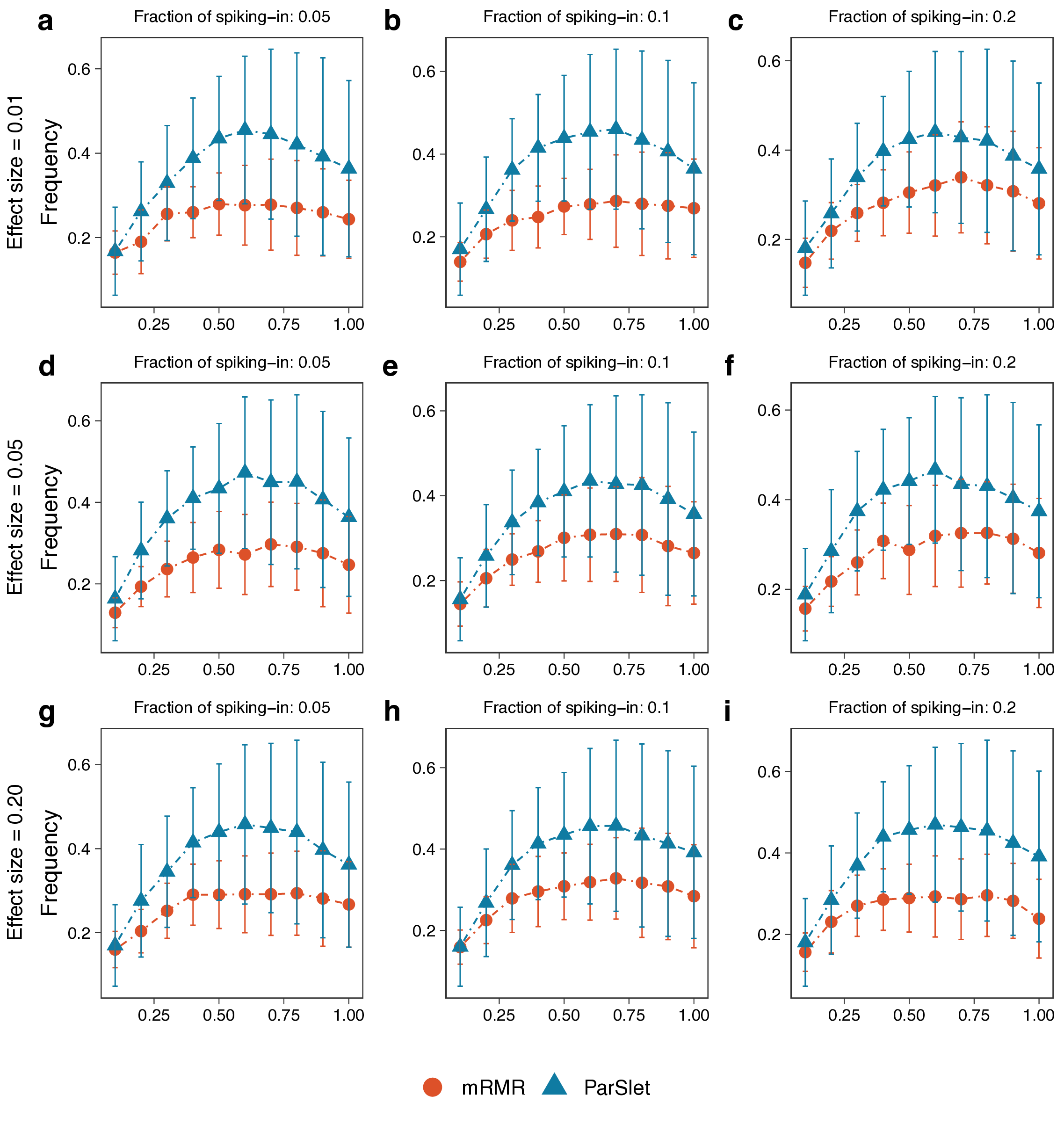
**

**Figure S15. Stability of feature selection under different effect sizes and spiking fractions using mRMR and ParSlet.** Each row corresponds to a different effect size (0.01, 0.05, 0.20). Each column corresponds to a different fraction of spiked-in features (0.05, 0.10, 0.20). The x-axis shows the fraction of samples used for training, and the y-axis shows the mean overlap of the top 10% ranked features between pairs of subsampled datasets. Red circles represent the standard mRMR ranking, while blue triangles represent the ParSlet ranking, in which feature scores are multiplied by prevalence raised to the power of 2.5.

**
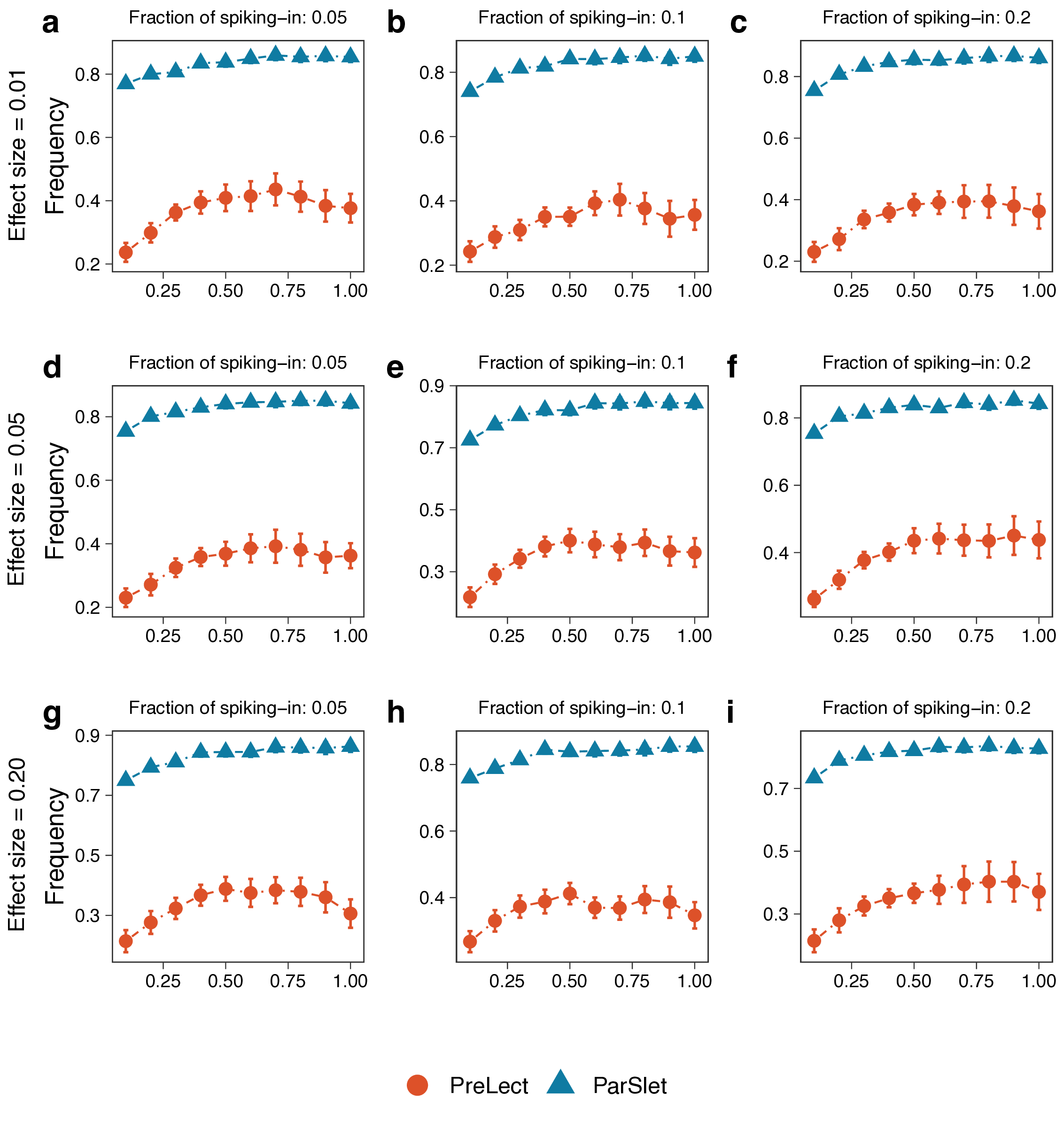
**

**Figure S16. Stability of feature selection under different effect sizes and spiking fractions using PreLect (λ = 30) and ParSlet.** Each row corresponds to a different effect size (0.01, 0.05, 0.20). Each column corresponds to a different fraction of spiked-in features (0.05, 0.10, 0.20). The x-axis shows the fraction of samples used for training, and the y-axis shows the mean overlap of the top 10% ranked features between pairs of subsampled datasets. Red circles represent the PreLect method, where the hyperparameter λ in PreLect was evaluated using a step size of 30; while blue triangles represent the ParSlet ranking (integrated Gini), in which feature scores are multiplied by prevalence raised to the power of 2.5.


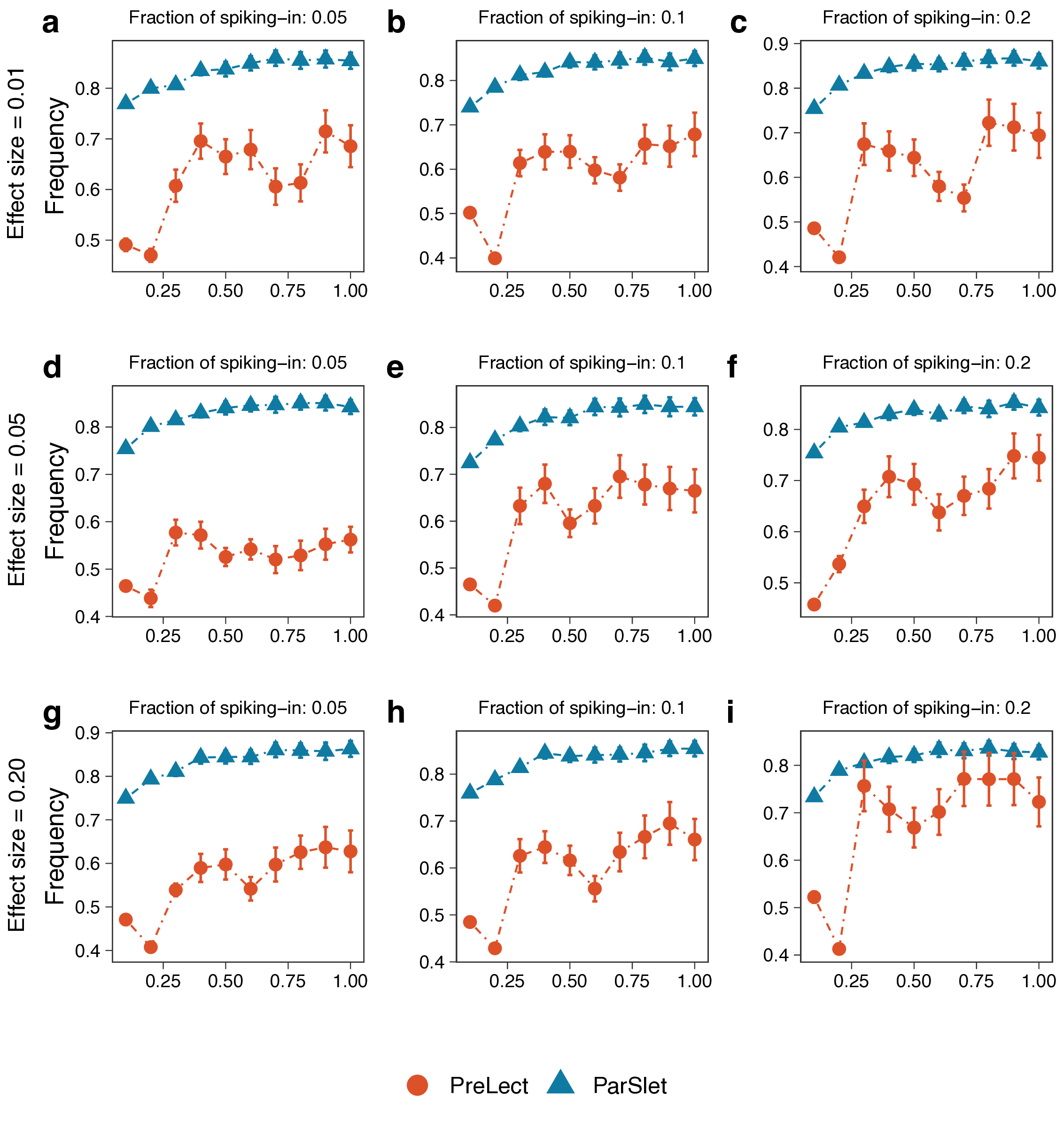


**Figure S17. Stability of feature selection under different effect sizes and spiking fractions using PreLect (λ = 10) and ParSlet.** Each row corresponds to a different effect size (0.01, 0.05, 0.20). Each column corresponds to a different fraction of spiked-in features (0.05, 0.10, 0.20). The x-axis shows the fraction of samples used for training, and the y-axis shows the mean overlap of the top 10% ranked features between pairs of subsampled datasets. Red circles represent the PreLect method, where the hyperparameter λ in PreLect was evaluated using a step size of 10; while blue triangles represent the ParSlet ranking (integrated Gini), in which feature scores are multiplied by prevalence raised to the power of 2.5.

**
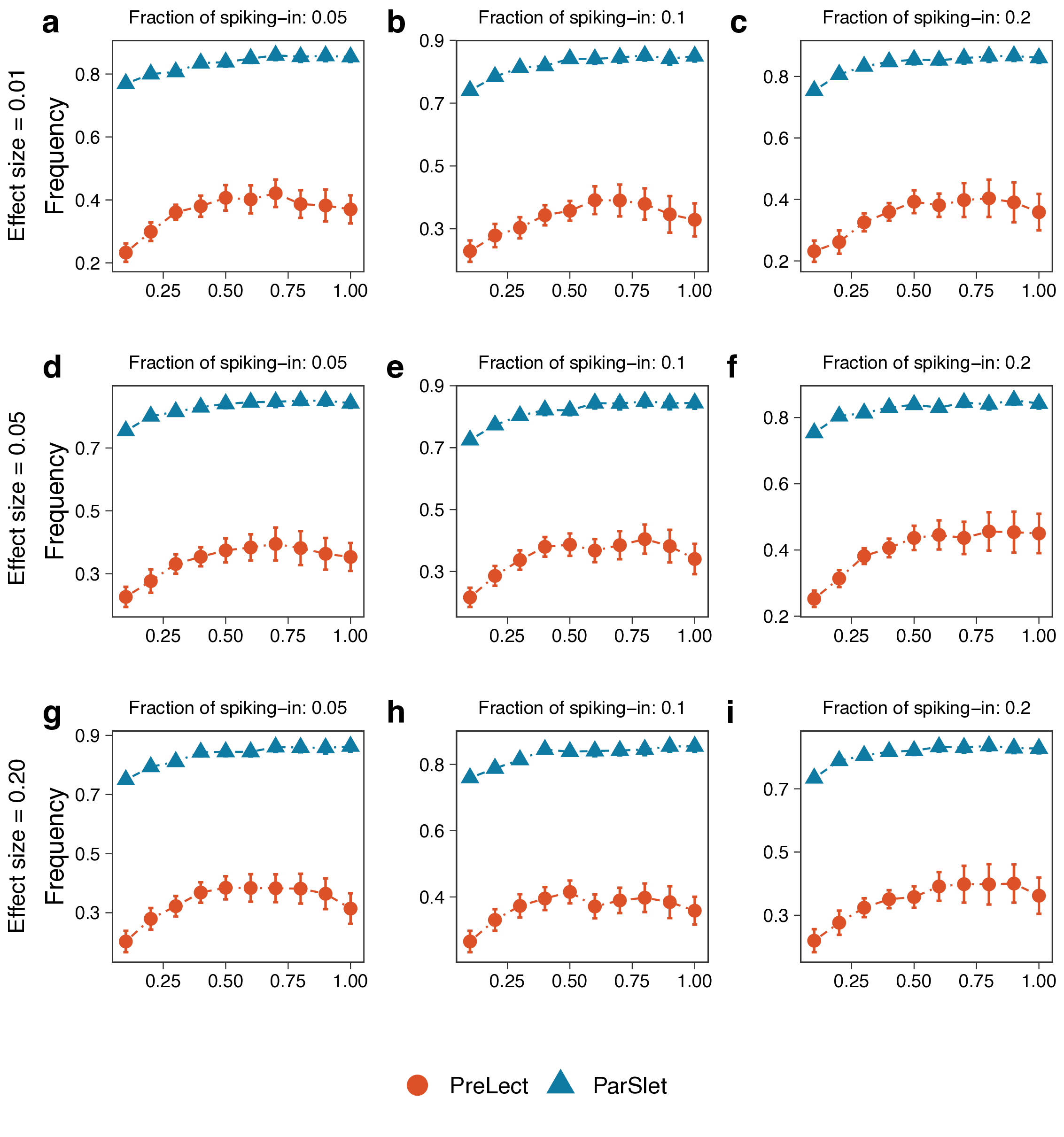
**

**Figure S18. Stability of feature selection under different effect sizes and spiking fractions using PreLect (λ = 50) and ParSlet.** Each row corresponds to a different effect size (0.01, 0.05, 0.20). Each column corresponds to a different fraction of spiked-in features (0.05, 0.10, 0.20). The x-axis shows the fraction of samples used for training, and the y-axis shows the mean overlap of the top 10% ranked features between pairs of subsampled datasets. Red circles represent the PreLect method, where the hyperparameter λ in PreLect was evaluated using a step size of 50; while blue triangles represent the ParSlet ranking (integrated Gini), in which feature scores are multiplied by prevalence raised to the power of 2.5.

**
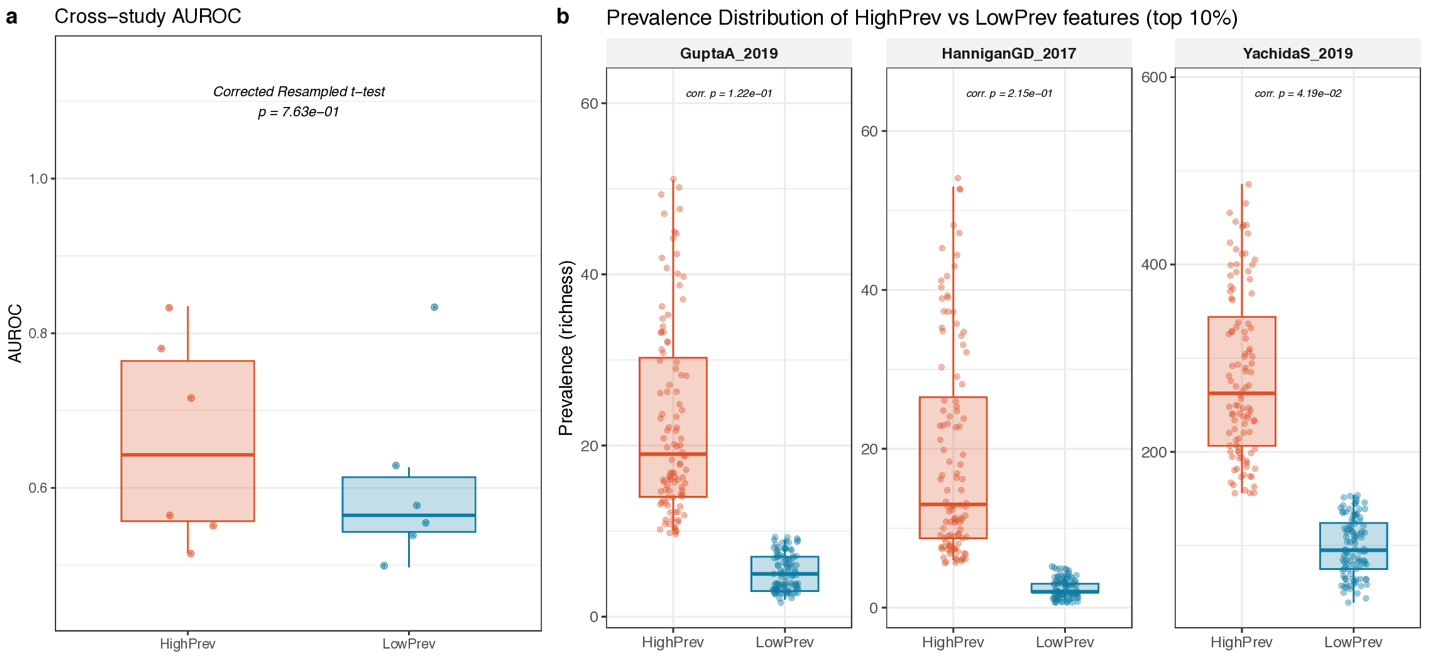
**

**Figure S19. Cross-study validation and prevalence stratification of selected features in colorectal cancer (CRC) cohorts. (a) Cross-study AUROC performance.** Random Forest classifiers were trained on one CRC cohort using feature selection performed exclusively within the training cohort and evaluated on an independent CRC cohort. Results are shown separately for feature sets derived from high-prevalence (HighPrev) and low-prevalence (LowPrev) groups based on the cohort-specific median prevalence. Each point represents the AUROC from one cross-study train-test pair, and boxplots indicate the median and interquartile range across all evaluations. Differences between high and low prevalence groups were computed using the corrected resampled t-test. (b) Prevalence distributions of HighPrev and LowPrev features. Boxplots show the distributions of feature prevalence (richness) with overlaid points representing individual features. Group differences were assessed using corrected resampled t-test, and corrected p-values are shown for each cohort.

**
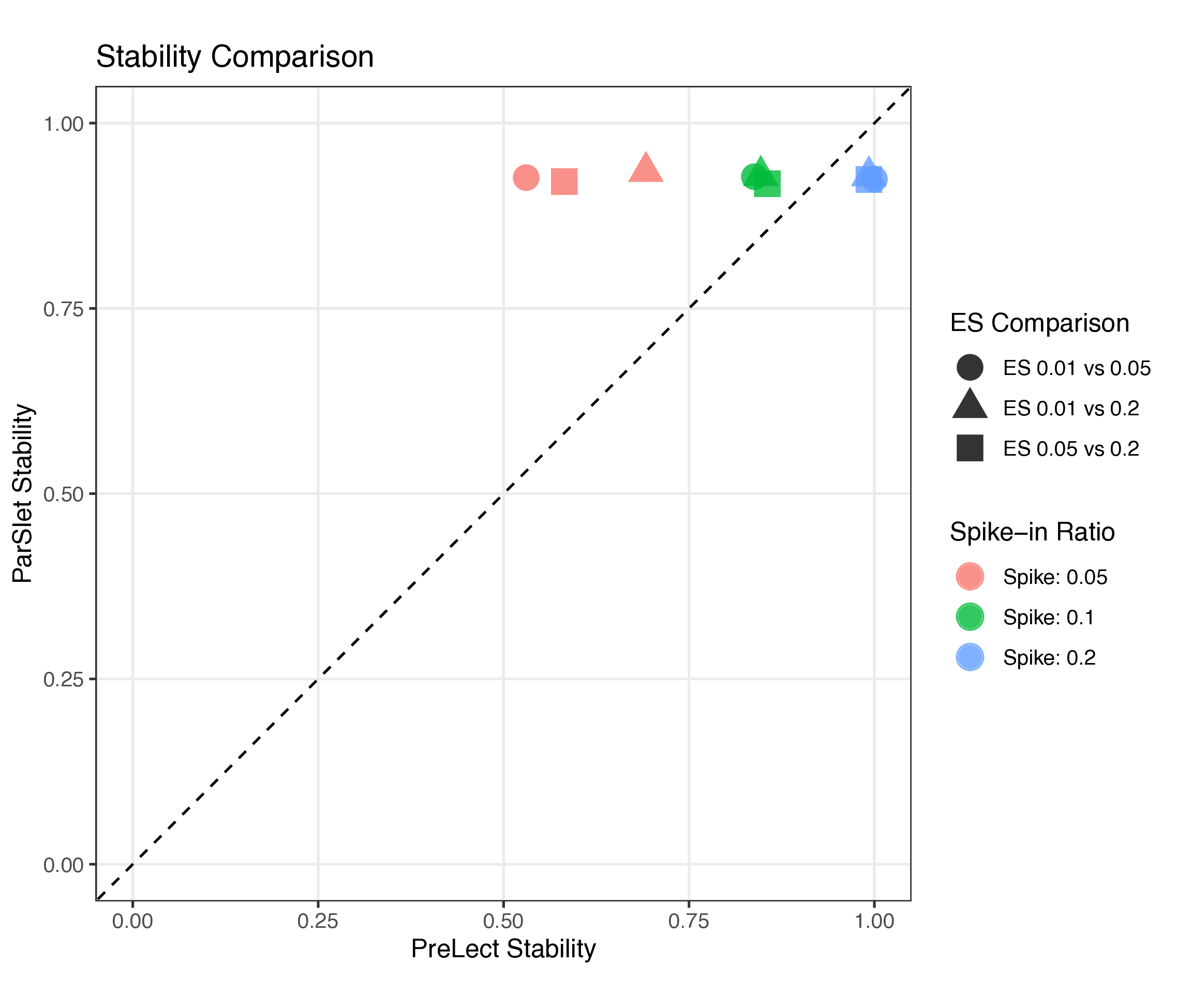
**

**Figure S20. Stability comparison between ParSlet and PreLect on simulated data.** Stability is defined as the Jaccard similarity between the sets of top selected features from pairwise comparisons of effect sizes (0.01 vs 0.05, 0.01 vs 0.2, and 0.05 vs 0.2), with ParSlet stability shown on the y-axis and PreLect stability on the x-axis. For each combination of spike-in fraction and effect size, models are trained on the full dataset and repeated ten times to account for stochasticity in the Random Forest model. Stability is computed by averaging the Jaccard similarity across these ten replicates for each pairwise effect size comparison. Each point represents a specific spike-in fraction and effect-size comparison. The dashed diagonal indicates equal performance; points above the line indicate higher stability for ParSlet.

**
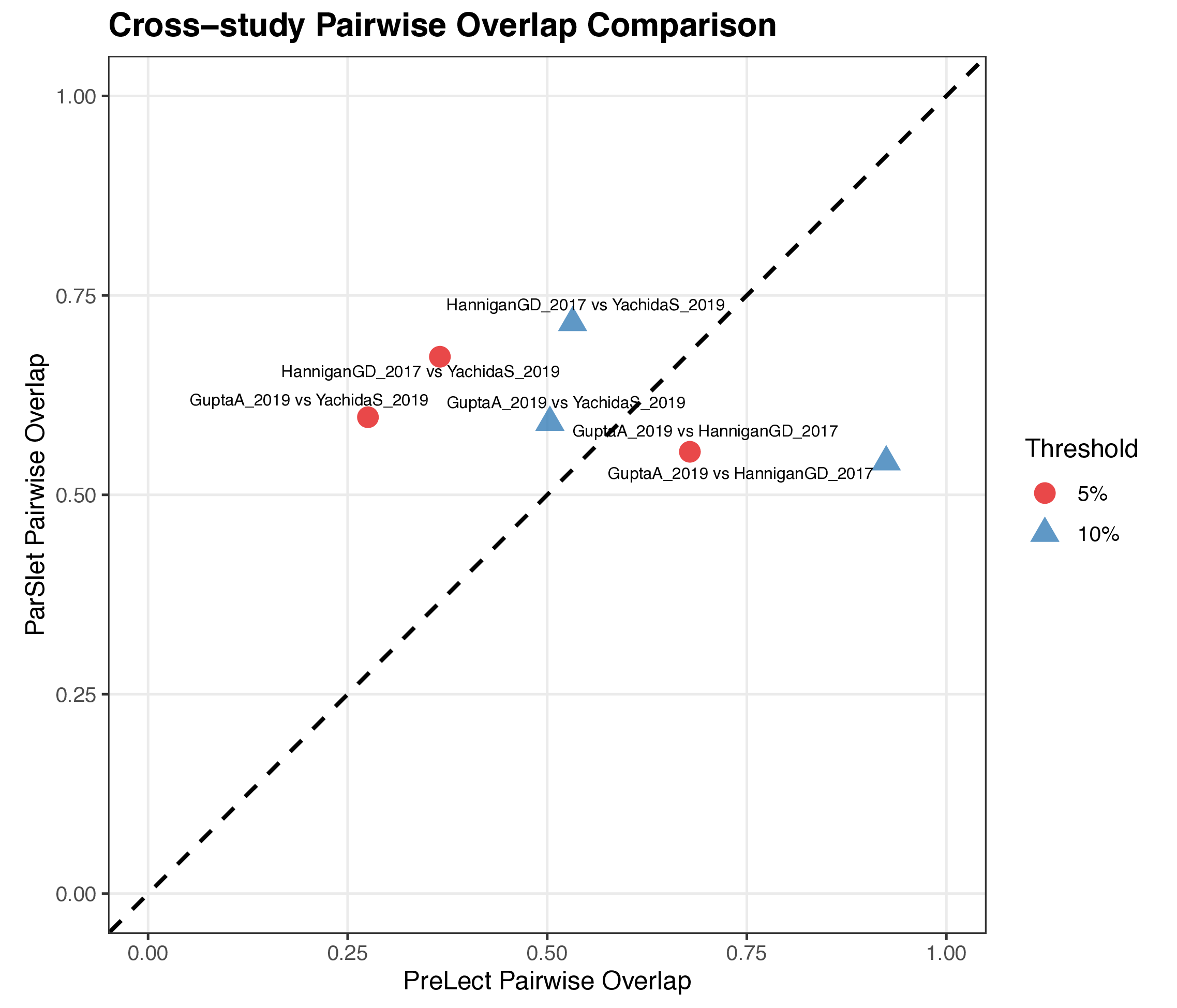
**

**Figure S21. Pairwise overlap comparison between ParSlet and PreLect in colorectal cancer (CRC) cohorts.** Overlap is defined as the proportion of shared features among the top 5% (red circles) or 10% (blue triangles) ranked features in each study pair, with ParSlet overlap shown on the y-axis and PreLect overlap on the x-axis. For each CRC study, feature selection was performed using all available samples (CRC cases and healthy controls) within that study. Each method was run once per study without repeated runs across multiple random seeds. The dashed diagonal indicates equal performance; points above the line indicate higher stability for ParSlet.

**
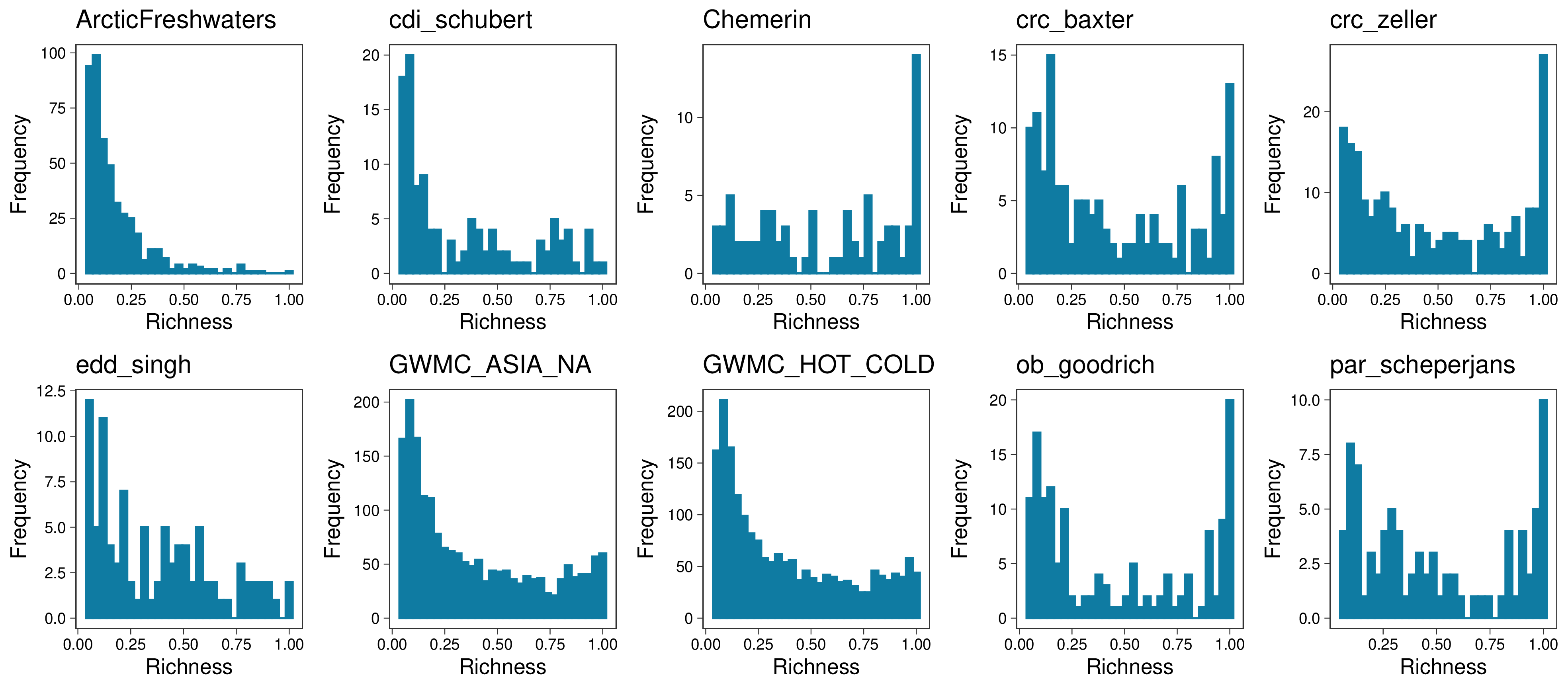
**

**Figure S22. Richness distribution of microbial features across real microbiome datasets.** This figure presents the richness distribution of microbial taxa in 10 real-world microbiome datasets. Richness is calculated as the proportion of samples in which a given taxon is present. Each panel corresponds to one dataset and displays a histogram of feature prevalence across samples. Most datasets exhibit a skewed distribution, with a large proportion of features having low prevalence (i.e., present in few samples), while a smaller number of features are widely present.

**Table S1. Top overlapped taxa and their frequencies among the top 10% important features identified by random forest from two colorectal cancer (CRC) datasets.**

| Taxon | Frequency  (original) | Frequency  (ParSlet) | Frequency  (LASSO) | Frequency  (Enet) | Frequency  (Relief) | Frequency  (mRMR) | Frequency  (PreLect) |
| --- | --- | --- | --- | --- | --- | --- | --- |
| *Ruminococcus* | 3 | 5 | 0 | 0 | 1 | 2 | 1 |
| *Clostridium_XVIII* | 4 | 5 | 0 | 0 | 1 | 1 | 3 |
| *Faecalibacterium* | 2 | 3 | 0 | 0 | 0 | 2 | 3 |
| *Blautia* | 4 | 5 | 0 | 0 | 0 | 0 | 1 |

**Table S2. Retained curatedMetagenomicData studies and baseline characteristics.**

| **Study** | **Phenotype** | **Cases (n)** | **Controls (n)** | **Total (n)** | **Country** | **Platform** | **Age (Med)** | **BMI (Med)** |
| --- | --- | --- | --- | --- | --- | --- | --- | --- |
| YachidaS_2019 | CRC | 258 | 246 | 504 | JPN | Illumina HiSeq | \| 64 \| \| --- \| | \| 22.5 \| \| --- \| |
| GuptaA_2019 | CRC | 30 | 30 | 60 | IND | Illumina NextSeq | \| 57 \| \| --- \| | \| 20.8 \| \| --- \| |
| HanniganGD_2017 | CRC | 27 | 28 | 55 | CAN; USA | Illumina HiSeq | \| 59 \| \| --- \| | \| 27.0 \| \| --- \| |
| FengQ_2015 | CRC | 13 | 16 | 29 | AUT | Illumina HiSeq | \| 65 \| \| --- \| | \| 23.2 \| \| --- \| |
| ThomasAM_2018a | CRC | 15 | 14 | 29 | ITA | Illumina HiSeq | \| 66 \| \| --- \| | \| 24 \| \| --- \| |
| HMP_2019_ibdmdb | IBD | 1201 | 426 | 1627 | USA | Illumina HiSeq | \| 21 \| \| --- \| | \| 21.7 \| \| --- \| |
| NielsenHB_2014 | IBD | 148 | 248 | 396 | DNK; ESP | Illumina HiSeq | NA | 25.4 |
| HallAB_2017 | IBD | 185 | 74 | 259 | USA | Illumina HiSeq | \| NA \| \| --- \| | \| NA \| \| --- \| |
| IjazUZ_2017 | IBD | 56 | 38 | 94 | GBR | Illumina HiSeq | \| 13 \| \| --- \| | \| NA \| \| --- \| |
| MetaCardis_2020_a | IGT | 52 | 234 | 286 | DEU; DNK; FRA | Ion Proton | \| NA \| \| --- \| | \| 29.6 \| \| --- \| |
| KarlssonFH_2013 | IGT | 49 | 43 | 92 | Multiple EU | Illumina HiSeq | \| 70 \| \| --- \| | \| 26.6 \| \| --- \| |
| MetaCardis_2020_a | T2D | 550 | 234 | 784 | DEU; DNK; FRA | Ion Proton | NA | \| 29.6 \| \| --- \| |
| KarlssonFH_2013 | T2D | 53 | 43 | 96 | Multiple EU | Illumina HiSeq | 70 | 26.6 |
| QinJ_2012 | T2D | 170 | 193 | 363 | CHN | Illumina HiSeq | NA | 23.8 |
| \| Sankaranarayanan_2015 \| \| --- \|  \|  \| \| --- \| | T2D | 19 | 18 | 37 | USA | Illumina HiSeq | 54 | 33.4 |
| YachidaS_2019 | Adenoma | 67 | 246 | 313 | JPN | Illumina HiSeq | 64 | 22.5 |
| HanniganGD_2017 | Adenoma | 26 | 28 | 54 | CAN; USA | Illumina HiSeq | 59 | 27.0 |
| ThomasAM_2018a | Adenoma | 16 | 14 | 30 | ITA | Illumina HiSeq | 66 | 24 |
| FengQ_2015 | Adenoma | 8 | 16 | 24 | AUT | Illumina HiSeq | 65 | 23.2 |
